# An infant burial from Arma Veirana in northwestern Italy provides insights into funerary practices and female personhood in early Mesolithic Europe

**DOI:** 10.1038/s41598-021-02804-z

**Published:** 2021-12-14

**Authors:** Jamie Hodgkins, Caley M. Orr, Claudine Gravel-Miguel, Julien Riel-Salvatore, Christopher E. Miller, Luca Bondioli, Alessia Nava, Federico Lugli, Sahra Talamo, Mateja Hajdinjak, Emanuela Cristiani, Matteo Romandini, Dominique Meyer, Danylo Drohobytsky, Falko Kuester, Geneviève Pothier-Bouchard, Michael Buckley, Lucia Mancini, Fabio Baruffaldi, Sara Silvestrini, Simona Arrighi, Hannah M. Keller, Rocío Belén Griggs, Marco Peresani, David S. Strait, Stefano Benazzi, Fabio Negrino

**Affiliations:** 1 Department of Anthropology, University of Colorado Denver, 1200 Larimer Street, Denver, CO 80217-3364 USA; 2 Department of Cell and Developmental Biology, University of Colorado School of Medicine, Aurora, CO 80045 USA; 3 Institute of Human Origins, School of Human Evolution and Social Change, Arizona State University, PO Box 2402, Tempe, AZ 85287-2402 USA; 4 Département d’anthropologie, Université de Montréal, Succ. Centre-Ville, CP 6128, Montréal, QC H3C 3J7 Canada; 5 Institute for Archaeological Sciences and Senckenberg Centre for Human Evolution and Paleoenvironment, University of Tübingen, Rümelinstr. 23, 72070 Tübingen, Germany; 6 SFF Centre for Early Sapiens Behaviour (SapienCE), University of Bergen, Øysteinsgate 3, Post Box 7805, 5020 Bergen, Norway; 7 Service of Bioarchaeology, Museum of Civilizations, Rome, Italy; 8 Department of Cultural Heritage, University of Padua, 35139 Padua, Italy; 9 Department of Cultural Heritage, University of Bologna, Via degli Ariani 1, 48121 Ravenna, Italy; 10 Skeletal Biology Research Centre, School of Anthropology and Conservation, University of Kent, Canterbury, CT2 7NZ UK; 11 DANTE Diet and Ancient Technology Laboratory, Department of Oral and Maxillo-Facial Sciences, Sapienza University of Rome, Rome, Italy; 12 Department of Chemical and Geological Sciences, University of Modena and Reggio Emilia, Modena, Italy; 13 Department of Chimistry G. Ciamician, Alma Mater Studiorum, University of Bologna, Via Selmi 2, 40126 Bologna, Italy; 14 Department of Human Evolution, Max Planck Institute for Evolutionary Anthropology, Deutscher Platz 6, 04103 Leipzig, Germany; 15 Ancient Genomics Laboratory, Francis Crick Institute, 1 Midland Road, London, NW1AT UK; 16 Department of Evolutionary Genetics, Max Planck Institute for Evolutionary Anthropology, 04103 Leipzig, Germany; 17 Cultural Heritage Engineering Initiative (CHEI), University of California San Diego, La Jolla, CA 92093 USA; 18 Department of Earth and Environmental Sciences, The University of Manchester, Oxford Rd, Manchester, M13 9PL UK; 19 Elettra - Sincrotrone Trieste S.C.P.A., 34149 Basovizza, Trieste Italy; 20 LINXS –Lund Institute of Advanced Neutron and X-ray Science, 223 70 Lund, Sweden; 21 Medical Technology Laboratory, IRCCS Istituto Ortopedico Rizzoli, Via di Barbiano 1/10, 40136 Bologna, Italy; 22 Department of Anthropology, Yale University, 10 Sachem Street, New Haven, CT 06511 USA; 23 Dipartimento Di Studi Umanistici, Sezione Di Scienze Preistoriche E Antropologiche, University of Ferrara, Corso Ercole I d’Este, 3244121 Ferrara, Italy; 24 Institute Environmental Geology and Geoengineering—IGAG CNR, 20131 Milan, Italy; 25 Department of Anthropology, Washington University, 1 Brookings Drive, St. Louis, MO USA; 26 Palaeo-Research Institute, University of Johannesburg, Cnr Kingsway and University Road Auckland Park, PO Box 524, Auckland Park, 2006 South Africa; 27 Department of Antiquities, Philosophy, History, University of Genoa, Via Balbi 2, 16136 Genoa, Italy

**Keywords:** Anthropology, Archaeology

## Abstract

The evolution and development of human mortuary behaviors is of enormous cultural significance. Here we report a richly-decorated young infant burial (AVH-1) from Arma Veirana (Liguria, northwestern Italy) that is directly dated to 10,211–9910 cal BP (95.4% probability), placing it within the early Holocene and therefore attributable to the early Mesolithic, a cultural period from which well-documented burials are exceedingly rare. Virtual dental histology, proteomics, and aDNA indicate that the infant was a 40–50 days old female. Associated artifacts indicate significant material and emotional investment in the child’s interment. The detailed biological profile of AVH-1 establishes the child as the earliest European near-neonate documented to be female. The Arma Veirana burial thus provides insight into sex/gender-based social status, funerary treatment, and the attribution of personhood to the youngest individuals among prehistoric hunter-gatherer groups and adds substantially to the scant data on mortuary practices from an important period in prehistory shortly following the end of the last Ice Age.

## Introduction

Mortuary practices offer a window into the worldviews and social structure of past societies. Ethnographically, many cultures have delayed attribution of personhood to young children, holding them in a liminal state of humanity^1–4^. Thus, child funerary treatment provides important insights into who was considered a person and thereby afforded the attributes of an individual self, moral agency, and eligibility for group membership. Indeed, significant discussion persists concerning the recognition of infant personhood among prehistoric peoples^5–7^. Here we report the burial of a young infant in Liguria (northwestern Italy)—Arma Veirana Hominin 1 (AVH-1; nicknamed “Neve”), directly dated to the early Holocene. In Europe, the onset of the Holocene (at 11,700 cal BP) broadly coincided with the early Mesolithic, a cultural period likely to have catalyzed important social changes as humans adapted to significant environmental shifts following the end of the last Ice Age^8,9^. Burials from the early Mesolithic are exceedingly rare or minimally documented^10–14^, and AVH-1 contributes essential data from this key period of prehistory. Significantly, AVH-1 represents the earliest female near-neonate interment documented in Eurasia and provides novel insights into how age and sex/gender influenced the construction of personhood among prehistoric hunter-gatherer societies.

## Results

**Figure 1:**
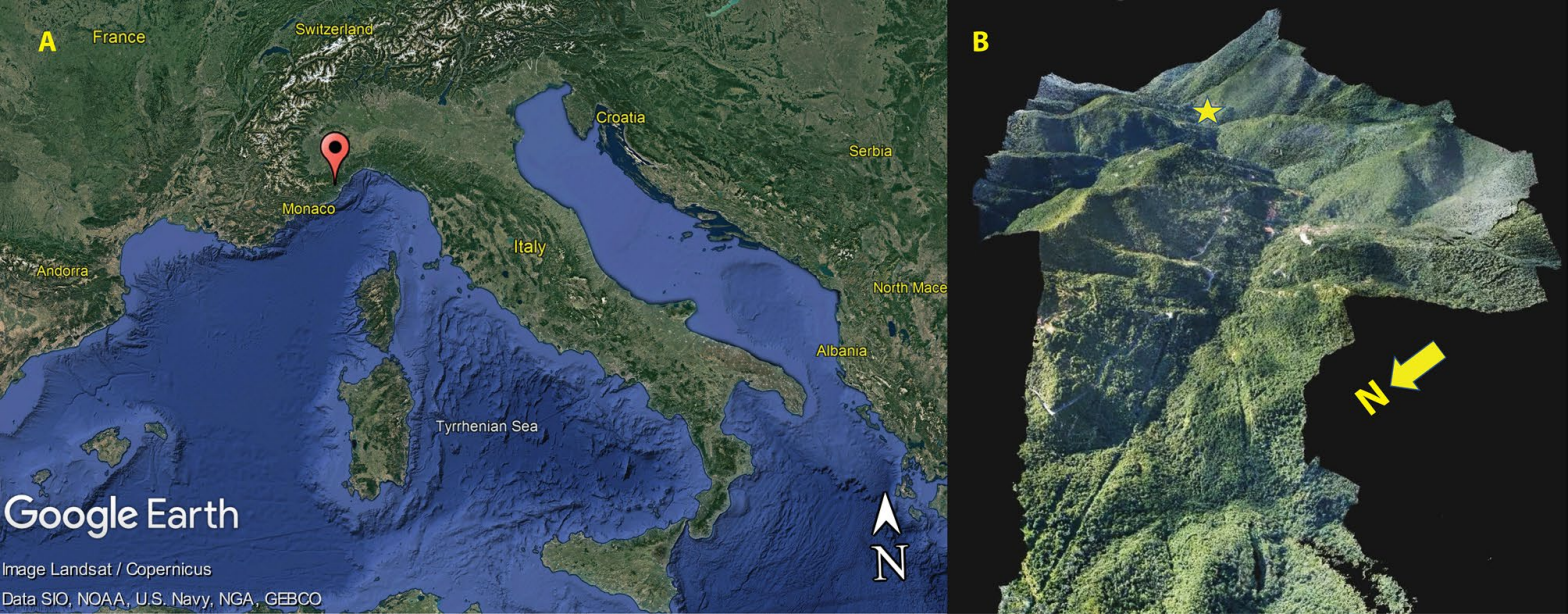
Location and setting of the site of Arma Veirana. (**A**) The red pin indicates the location of Arma Veirana located in the Val Neva (Neva River Valley) within the region of Liguria (northwestern Italy)—map made using Google Earth Pro 7.3.4 (https://earth.google.com); (**B**) 3D photogrammetric model of the Val Neva in the Ligurian Pre-Alps generated from aerial photography by coauthor DM (star = location of Arma Veirana).

**Figure 2:**
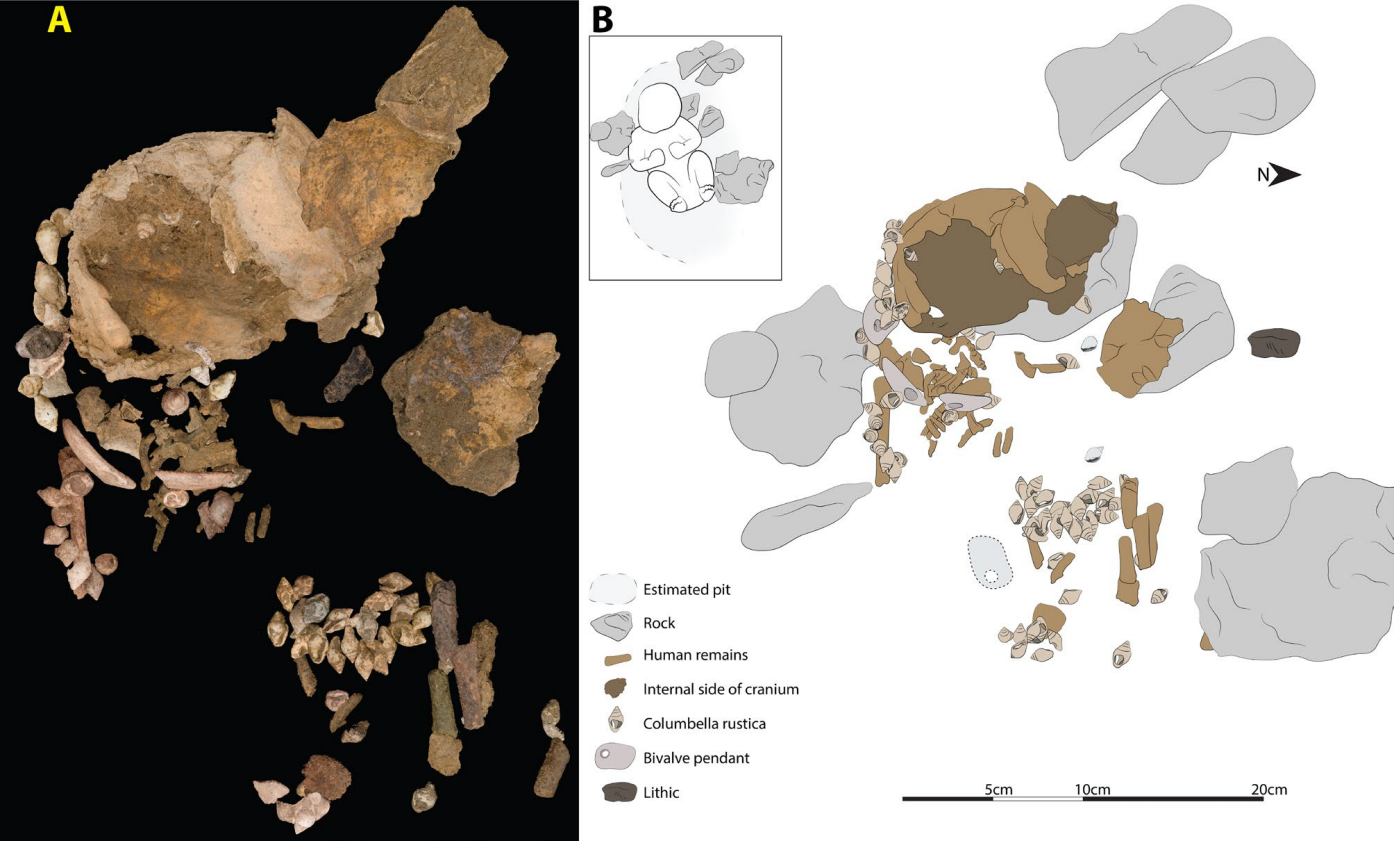
Burial layout. (**A**) Progressive 3D photogrammetric image of each piece prior to removal, reconstructing the bones and artifacts as they were in situ. (**B**) Tracing with inset demonstrating the likely body position. Movie S1 shows a fly-through of a full 3D photogrammetric model of Arma Veirana (created by coauthors DM, DD, and FK), and an interactive 3D model of the cave is available online: https://bit.ly/3jCq4zC.

Arma Veirana is located within the Ligurian pre-Alps (Fig. 1) approximately 15 km northwest of the town of Albenga (44°8′45.402″N, 8°4′18.85E). The cave is situated on the northside of the steep-sided Val Neva within a marble cliff-face at an elevation of 451 m above sea level. Arma Veirana preserves deposits of late Pleistocene (with Mousterian and late Epigravettian cultural horizons) and early Holocene age. The Supplementary Information includes additional details on site location, stratigraphy, and geoarchaeology, along with site plan maps and the history of excavation. In 2017 and 2018, in situ skeletal remains (Table S8) and associated artifacts were recovered within a 15 cm deep oval pit (< 800 cm^2^ in area) cut into the underlying late Epigravettian deposit (Figs. 2, S9, S10). Movie S1 shows a fly-through of a 3D photogrammetric model of the cave, including the position of the burial excavation.

### Skeletal remains, radiocarbon age, and biological profile

The neurocranium, articulated right scapula and humerus, and articulated ribs and thoracic vertebrae indicate a supine body position with head to the west and lower limbs to the east (Fig. 2). AMS radiocarbon dating of a vertebral arch provides a direct age of 10,211–9914 cal BP (95.4% probability; Table S7).

An inventory of skeletal remains is provided in Table S9 and the 3D coordinates for the plotted elements are included in Supplementary data file 1. The bony remains of the AVH-1 infant are heavily damaged due to surface trampling, water erosion, or chemical alteration. The shallow depth of the burial pit relative to the modern cave floor likely resulted in compression and fragmentation of much of the skeleton. Most of the mid-abdominal region of the skeleton is missing, including the caudal aspect of the thorax, lumbar region, and pelvis. Portions of the lower limb shaft fragments are positioned close to the likely anatomical position of the burial pose (Fig. 2) but are largely featureless and at least partly fossilized and concreted. Increased mineralization of these lower limb elements suggests some increased water movement through this portion of the burial pit.

Among the postcranial remains, the right humerus [Plotted Find (PF) #3855] and right scapula (PF#9245) are the most complete elements and their close association indicates that they were in approximate anatomical articulation with one another. Their positioning relative to the apparently articulated shell beads and pendants suggest little movement and likely represent the original location of the shoulder at the time of burial. A portion of the right upper thorax was mostly intact and in approximate anatomical position. Several ribs are preserved but they were exceptionally fragile and were heavily fragmented during the process of excavation; however, their original positioning is captured in the photogrammetric model (Fig. 2). The thorax location is consistent with the interpretation that the right shoulder is in its original in situ context at the time of interment.

Although crushed, much of the neurocranium is present, with most of the vault portions of the cranial base collected within a single block (Plotted Find #9131) along with disarticulated right (PF#4631) and left (PF#6969) frontal bones. Isolated and developmentally-unfused components of the occipital bone were collected separately (right pars lateralis PF#9241; left pars lateralis PF#6288). Other than the right hemimandible (PF#6273), the entirety of the viscerocranium (facial skeleton) is missing.

**Figure 3:**
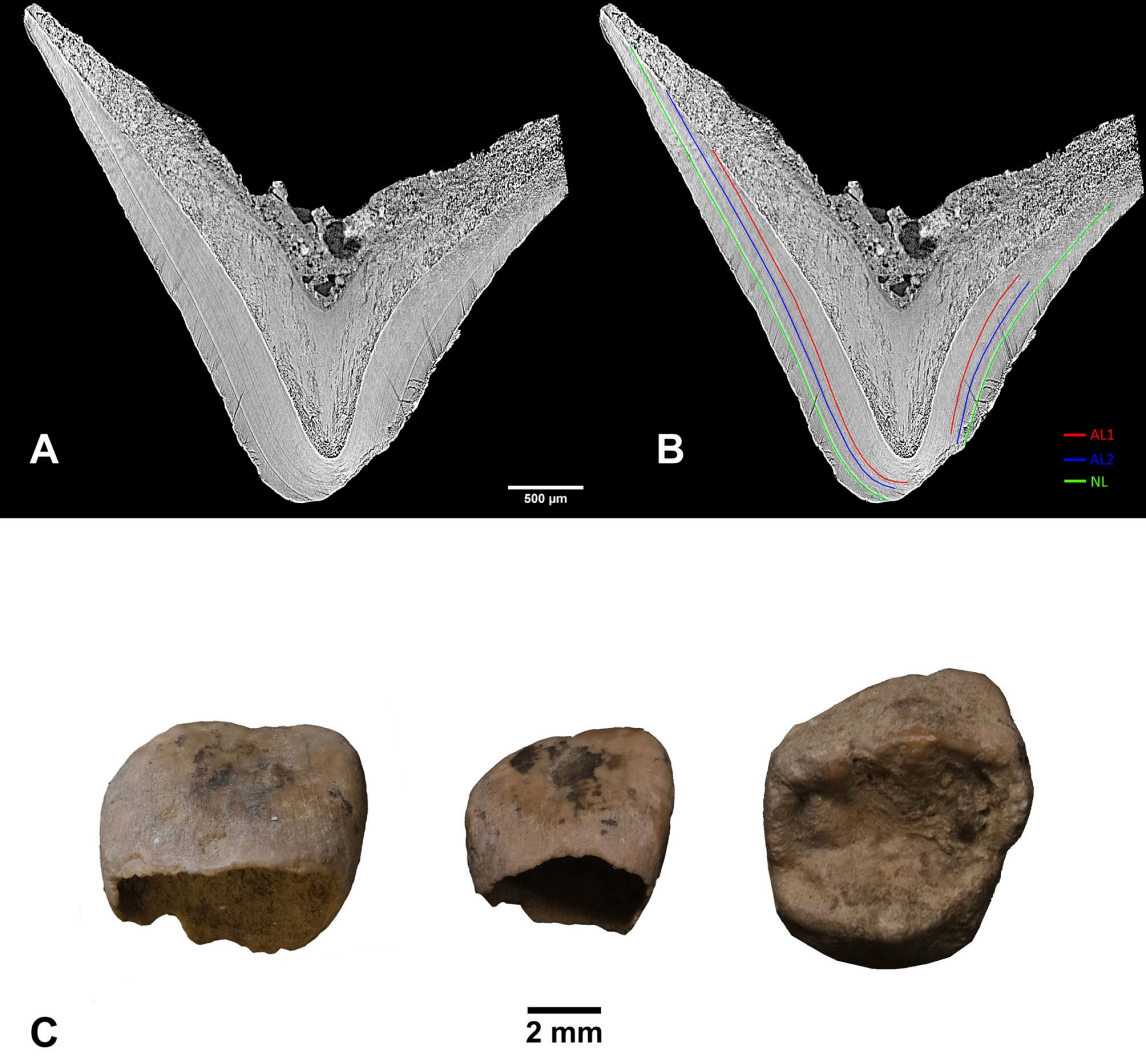
Virtual histology. (**A**) Vestibular-lingual section of the AVH-1g deciduous upper first molar passing through the mesio-lingual cusp (pixel size = 3 µm, reformatted slice thickness 30 µm); (**B**) The same section showing position of the neonatal line (NL in green) and two accentuated lines (AL1 & AL2 in red and blue, respectively). Position of the NL allowed estimation of AVH-1’s age at death based on enamel development. The two accentuated lines reflect prenatal stress events. (**C**) Three deciduous teeth investigated through synchrotron X-ray computed microtomography; from left to right: AVH-1d (upper central incisor, vestibular view), AVH-1c (upper lateral incisor, vestibular view), AVH-1g (upper first molar, occlusal view).

**Figure 4:**
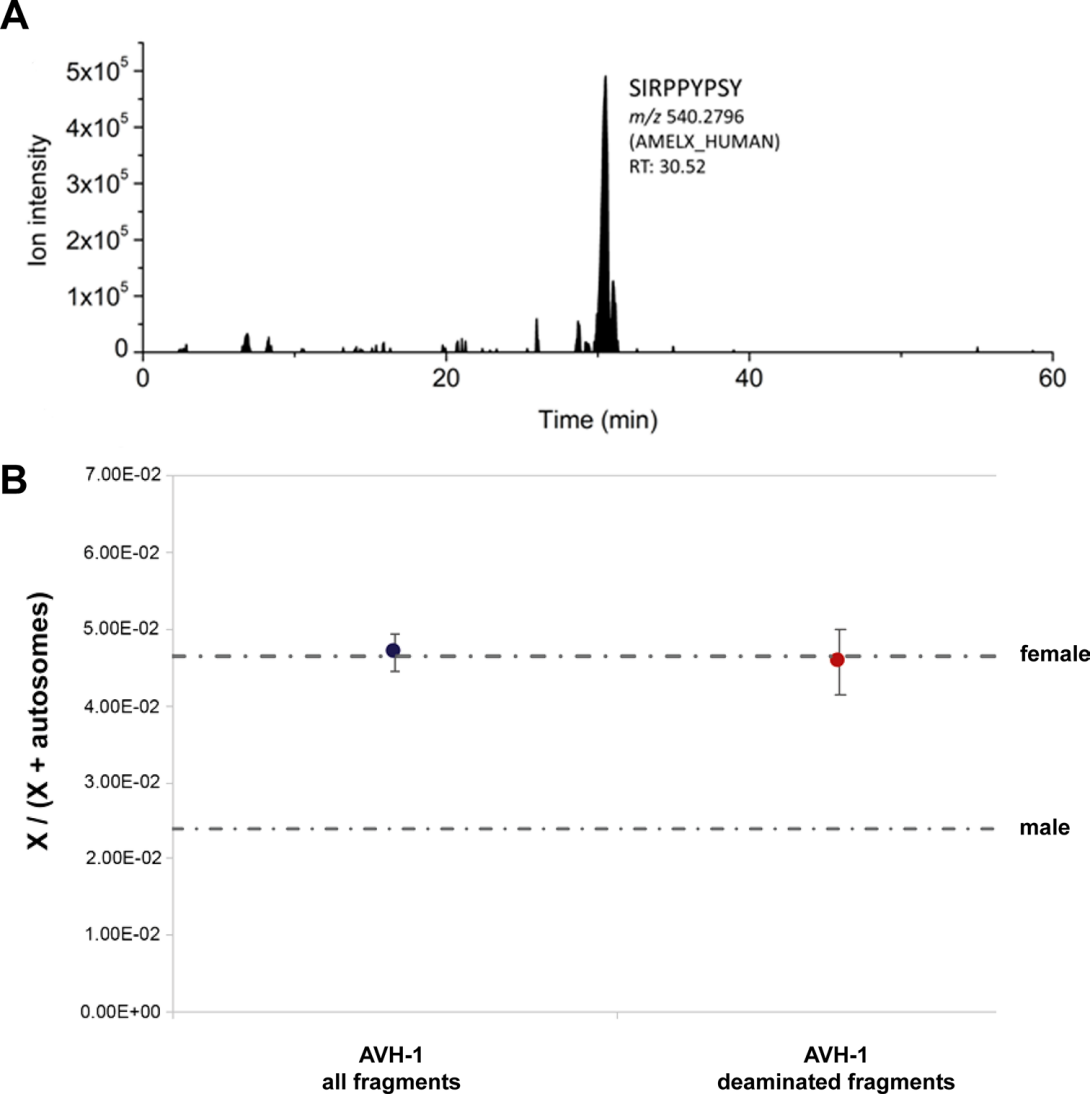
Sex determination of AVH-1 based on proteomics and number of nuclear DNA fragments aligning to the X chromosome and the autosomes. (**A**) Ion chromatograms of peptide SIRPPYPSY (AMELX, 540.2796 m/z) as in^22^; peptide SM(ox)IRPPY (AMELY) has not been detected (or any other AMELY peptide), reflecting female status; (**B**) Dashed lines represent the expected ratios of X to (X + autosomal) fragments for a female and a male. Results were concordant for all fragments (blue dot) and for deaminated fragments (red dot). Vertical bars depict the 95% binomial confidence intervals.

Gross tooth development indicates that AVH-1 was less than two months old (postnatal)^15^, but results from the virtual histological measurement (using synchrotron imaging) of postnatal enamel formation^16,17^ provides a more precise age-at-death of 40–50 days (Fig. 3; Table S11). Accentuated lines in prenatal enamel (Fig. 3B) also reveal stress episodes affecting the fetus or mother at approximately 47 and 28 days before birth. Stable isotopes of collagen (δ^13^C and δ^15^N; Table S7)—possibly not yet affected by breastfeeding signals^18,19^—suggest a terrestrial diet for the mother of AVH-1 similar to late Upper Palaeolithic individuals from the area^20,21^. Proteomic analysis (Fig. 4A) demonstrates a lack of AMELY protein and therefore likely attribution to female sex^22,23^, a result that is confirmed by a count of nuclear DNA fragments that align with the X chromosome and autosomes (Fig. 4B). The mtDNA molecular age AVH-1 is estimated at 9,774 years BP (95% highest posterior density: 1,853–16,662), similar to the radiocarbon age. AVH-1’s mtDNA haplogroup is U5b2b and it nests within a clade of late Pleistocene and early Holocene individuals from central and western Europe (Fig. S13).

### Ornaments and other artifacts

**Figure 5:**
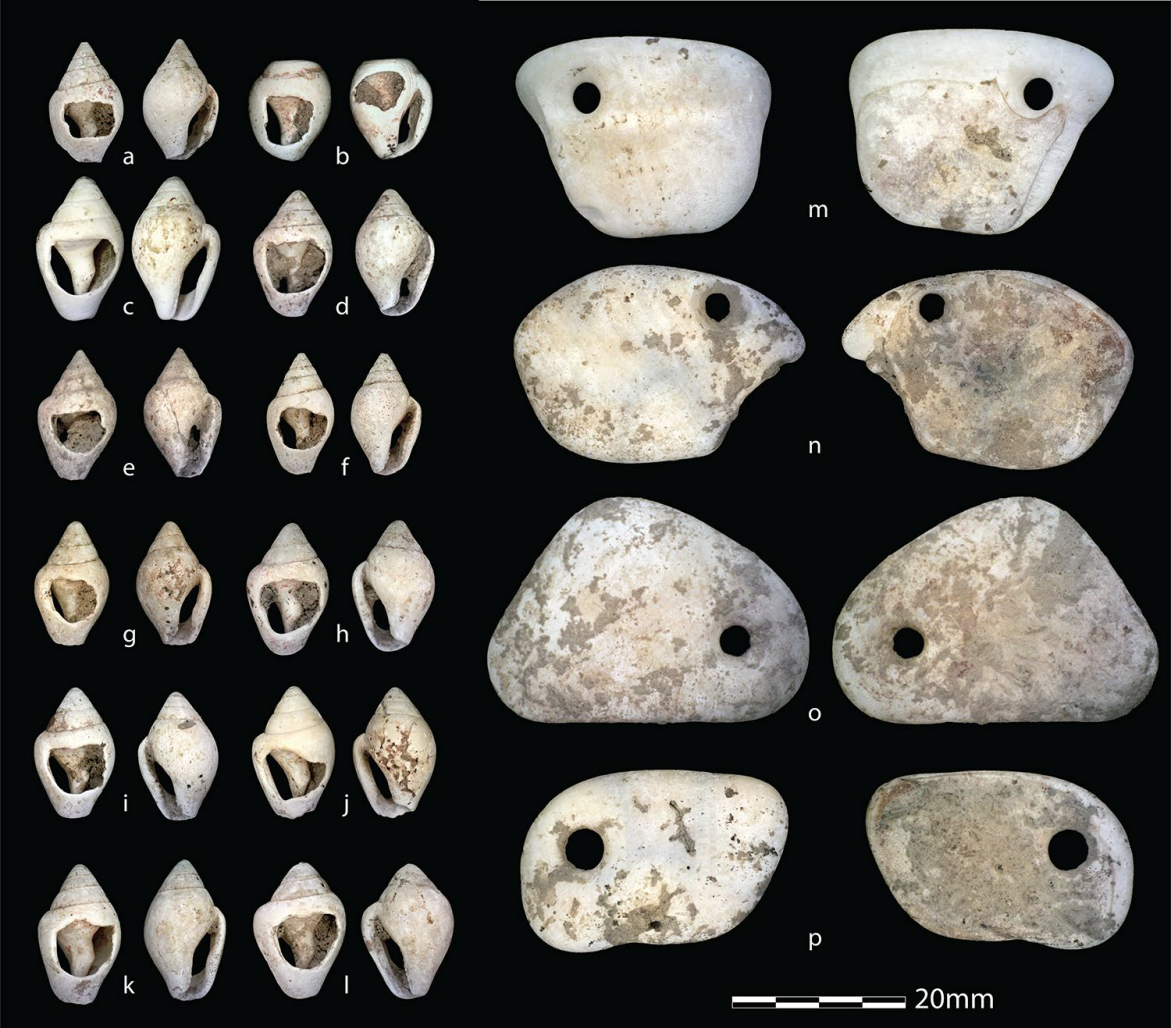
Ornaments associated with AVH-1. Examples of *Columbella rustica* shell beads (**a–l**) and pierced pendants made from *Glycmeris* sp. (**m–p**).

AVH-1 was adorned with at least 66 perforated *Columbella rustica* ornamental shell beads and three perforated pendants made on polished fragments of *Glycymeris* sp. (Fig. 5). A line of shell beads and three pendants was recovered in situ over the right shoulder and upper thoracic region (Fig. 2), suggesting they were sewn onto a blanket or hood. Over 20 of the *C. rustica* beads covered the abdominal region (Fig. 2), possibly reflecting a beaded vestment or other item skirting the waist and torso. In addition to the beads closely associated with the skeleton, 27 *C. rustica* beads, one *Turritella* sp., and one *Glycymeris* pendant come from pit fill or disturbed contexts nearby. Most shell beads bear significant wear, implying a lengthy use-life, and were probably not made originally for funerary purposes; rather, the infant likely received beads initially worn by other individuals. Regardless, they represent significant labor. Preliminary experiments estimate manufacture of all the ornaments required 8–11 person hours, not including time needed to collect shells and sew the beads onto a garment.

Other artifacts associated with AVH-1 include a claw (pedal phalanx) of an eagle-owl (*Bubo bubo*) recovered ~ 20 cm from the infant remains in what may have been a connected secondary pit. Surface modifications are consistent with ornamental use (Figs. S14, S15). A gray flint laminar flake with marginal, direct retouch (PF#4587) was collected near the cranium and other than the ornaments, is the artifact in closest spatial relationship to the child (Fig. S16). A fragmentary Sauveterrian point, common in the early Mesolithic^24^, but also occasionally found in the local Epigravettian^25^, comes from the top of the pit fill (Fig. S17). Five pieces of ochre and 19 flint and radiolarite debitage pieces were also recovered in pit fill (Table S13). Highly fragmented faunal remains of large and medium-sized artiodactyls, birds, and unidentifiable mammal bones (Tables S14 and S15) were recovered in the burial fill and adjacent sediments (see Supplementary Results).

Though the shell ornaments were clearly worn or otherwise covered the body, there is uncertainty as to whether the other artifacts were buried as grave goods or derive from earlier deposits into which the pit was dug and then refilled. Radiocarbon dates from charcoal and faunal fragments indicate dates of 15,000 – 16,000 calBP for the fill contents (see Table S8 and Fig. S11), suggesting an Upper Paleolithic (Late Epigravettian) deposit as the probable substrate into which the burial was dug. Thus, many of the lithics probably derive from the deposits underlying the burial. Given its close spatial relationship, the gray flint laminar flake is the most likely to be truly associated with the skeletal remains. The Sauveterrian point is the piece closest to being a diagnostic Mesolithic artifact; however, it was recovered from sieved material removed from a higher elevation within the burial fill. See further commentary on the lithics in the Supplementary Results.

## Discussion

The abundance of ornamental artifacts associated with AVH-1’s single, intentional burial suggests that at least some early Mesolithic groups attributed personhood to near-neonatal girls. Given the dearth of early Mesolithic samples in general, and infants in particular, it is difficult to generalize the results to other regions of Europe at the onset of the Holocene. However, the interment of female neonates and infants is demonstrated at later Mesolithic and early Neolithic sites such as Lepenski Vir in the Danube Gorges. Work at Lepenski Vir^26,27^ on a sample of 40 young infants has included DNA-based sex identification^28^ and shown that young female were buried in similar frequencies to their male counterparts with no strong pattern differentiating sexes on the basis of spatial distribution or grave goods (e.g., beads).

Unfortunately, for most Mesolithic and Neolithic sites, exact age and sex data are unavailable for the infant samples, leaving much to be understood about how these variables intersect with the construction of personhood. At least some southern European sites preserving the later Mesolithic and Neolithic have been argued to show that young children were often treated similarly to adults in terms of burial rites^29^, suggesting a general attribution of personhood to young individuals. The relatively large later Mesolithic cemeteries of northern Europe also included significant numbers of child inhumations^14,30^. In some late Mesolithic cases, young infants (< 1 year of age) were provided apparently symbolic treatments, such as seen with the perinatal child interred with (presumably) its mother at Vedbaek in Denmark. The young Vedbaek child was laid upon a swan’s wing and covered in ochre^31^, suggesting some degree of at least lineage-imparted social status. However, there is considerable spatial and temporal variation in infant funerary treatment across the late Mesolithic of northern Europe^14,30^. Children at sites such as Skateholm in Sweden who were interred separately from adults were mostly buried without grave artifacts^32^. The well-preserved child (< 6 months of age) from the site of Groß Fredenwalde in Germany dated to approximately 8400 calBP^33^ is likely to provide important insights (especially when a full biological profile has been completed) by pushing back the northern European infant record earlier into the Mesolithic.

Though infant burials are known from earlier (Middle and Upper Paleolithic and early Mesolithic) contexts in Europe and broader Eurasia^6,7,10–13^, most are from old excavations or otherwise minimally documented. Biological profiles including exact age-at-death and sex are thus unavailable in nearly all cases. A pair of neonates dated to 27,000 cal BP at the Gravettian site of Krems-Wachtberg^34^ is an important exception, shown to comprise male monozygotic twins by the analysis of ancient DNA^35^. However, a < 6 week old DNA-confirmed female infant interred with grave goods from Upper Sun River in Eastern Beringea (11,500 cal BP) illustrates that mortuary treatment of baby girls similar to that observed at Arma Veirana characterized terminal Pleistocene cultures elsewhere^36,37^. This implies that infant personhood inclusive of females has deeper origins in a common ancestral culture or that it arose in parallel in nearly contemporaneous populations across the planet. Either way, the terminal Pleistocene and earliest Holocene should be considered the minimum antiquity for the recognition of young girls as members of society in cultures around the globe.

As delayed personhood often corresponds with high risk of infant death, the AVH-1 and Upper Sun River burials could suggest relatively low levels of infant mortality even as hunter-gatherers adapted to diverse geography and the shifting environments of the late Pleistocene and early Holocene. In some ethnographic examples, when personhood is attributed to infants, abundant grave ornaments served to bolster that status beyond death to compensate for the child’s fragility^38^. The extension of such treatment to female infants among early Holocene hunter-gatherers suggests a degree of egalitarian treatment of individuals in life and death regardless of age or sex/gender.

## Materials and methods

Fieldwork at Arma Veirana and all sampling of the AVH-1 skeleton and other artifacts was conducted under the auspices of Soprintendenza Archeologia, Belle Arti e Paesaggio per la città metropolitana di Genova e le province di Imperia, La Spezia e Savona via a permit formally issued to coauthor Fabio Negrino, following all relevant requirements of the permitting authority.

### Fieldwork and site documentation

Spatial relationships in the Arma Veirana excavation have been documented using total stations and photogrammetry to establish 3D coordinates for all artifacts recovered in situ (no size cutoff). Details concerning establishment of the excavation grid using control points (Fig. S1; Table S2) and other details are provided in the Supplementary Information. Standard field-based descriptions of deposits and stratigraphy were combined with micromorphology. Micromorphological analysis of intact blocks of sediment allowed for high resolution documentation of the burial’s geological context, including the composition and origin of the burial fill (see Supplementary Methods).

Excavation of the delicate AVH-1 burial was not conducted en bloc; due to the surroundings’ rugged terrain, transporting a burial block to the lab was not possible. Instead, each piece was removed on site. To document the 3D spatial relationships of skeletal remains and grave goods to one another, total station data were combined with a system of progressive photogrammetry (Fig S2). Further technical details are provided in the Supplementary Methods.

### Radiocarbon dating and carbon stable isotopes

With permission of the Soprintendenza Archeologia, Belle Arti e Paesaggio per la città metropolitana di Genova e le province di Imperia, La Spezia e Savona, and following all relevant guidelines issued by that authority, one human vertebral neural arch from the Arma Veirana burial (plotted find #9237; Fig. S3) was pretreated at the Department of Human Evolution at the Max Planck Institute for Evolutionary Anthropology, Leipzig, Germany, using the method described both in^39,40^ for bone samples < 200 mg: the outer surface of the bone sample was first cleaned by a shot blaster and the whole bone decalcified in 0.5 M HCl at room temperature until no CO_2_ effervescence was observed. 0.1 M NaOH was added for 30 min to remove humics, followed by a final 0.5 M HCl step for 15 min. The resulting solid was gelatinized at pH 3 in a heater block at 70 °C for 9 h and filtered in an Eeze-Filter™ (Elkay Laboratory Products (UK) Ltd.) to remove small (> 80 µm) particles. The gelatine was ultrafiltered^41^ with Sartorius “VivaspinTurbo” 30 KDa ultrafilters. Prior to use, the filter is cleaned to remove carbon containing humectants^42^. The samples were lyophilized for 48 h.

The collagen was weighed into a pre-cleaned tin cup and sent to the Curt-Engelhorn-Centre for Archaeometry Klaus-Tschira-AMS facility in Mannheim, Germany (lab code: MAMS) for graphitization and dating with the MICADAS-AMS^43^. To monitor contamination introduced during the pre-treatment stage, a sample from a cave bear bone, kindly provided by D. Döppes (MAMS, Germany), was extracted along with the batch AV1^44^. To assess the preservation of the collagen yield, C:N ratios, together with isotopic values must be evaluated. The C:N ratio should be between 2.9 and 3.6 and the collagen yield not less than 1% of the weight^45^. Stable isotopic analysis was conducted at MPI-EVA, Leipzig (Lab Code R-EVA), using a ThermoFinnigan Flash EA coupled to a Delta V isotope ratio mass spectrometer.

Several other samples collected in 2017 at the beginning of the burial excavation were dated at the University of Oxford Radiocarbon Acceleration Unit (ORAU) to provide broader contextual information to inform interpretations of the burial and site formation. These included faunal bone and charcoal samples that derive from the burial fill or adjacent sediments. The ORAU protocols include the correction of isotopic fractionation using δ^13^C values measured using the accelerator mass spectrometer, which are also reported independently using results from a stable isotope mass spectrometer (to ± 0.3 per mil relative to VPDB). Details of the ORAU protocols can be found elsewhere^46,47^.

### Dental analyses

#### Virtual histology

Human teeth permanently record growth information during their formation allowing assessment of tooth formation times, stresses experienced during development, and age-at-death (in infants). Dental ontogenetic studies rely on the rhythmical growth of enamel and dentine, producing short and long period incremental markings, visible in longitudinal thin sections of teeth^48^. The deposition of enamel and dentine matrix is subject to inner biological rhythms: a circadian growth process that produces prismatic cross striations (CS) in enamel and von Ebner’s lines in dentine, and a longer periodicity represented by Retzius lines in enamel and Andresen lines in dentine^16,49^. Stress events strong enough to disrupt development leave marks in the corresponding position of the enamel or dentine front, visible as Accentuated Lines (ALs)^49^. Usually, the first accentuated line, characterizing all the deciduous teeth and the first permanent molar of individuals that survive the perinatal stage, is the Neonatal Line (NL), which separates the tissues formed prenatally from that growing after birth^50^. ALs in the prenatal enamel are relatively rare and indicate physiological stresses affecting the mother or fetus^51^. The NL is used to calibrate the chronology of stresses and the time of pre- and postnatal crown growth. Histomorphometry of dental enamel allows the collection of parameters such as the Daily Secretion Rate (DSR, i.e. the speed at which the ameloblast—the enamel forming cells—moves towards the outer surface of the tooth) and, for still growing crowns, the age-at-death as the time spent from the NL to the end of enamel formation^16,51,52^.

Virtual histomorphometry of three deciduous tooth crowns (AVH-1d, left upper central deciduous incisor; AVH-1c, left upper lateral deciduous incisor; AVH-1g, right upper deciduous first molar) was conducted to identify the NL, assess chronological age-at-death, and investigate biological life history. Precautions were adopted to preserve ancient DNA^53^. The dental deciduous crowns were imaged through synchrotron radiation computed microtomography (SRμCT)^17,51,54^ at the SYRMEP beamline of the Elettra Sincrotrone Trieste laboratory in Basovizza (Trieste, Italy)^55^. Details of the scanning protocol are provided in the supplementary materials.

Virtual histological sections passing through the bucco-lingual plane at the tip of the dentine horn of the incisors and of the mesio-lingual cusp (protocone) of AVH-1g were derived from the synchrotron radiation computed microtomography volumes (Figs. 2A, S4, S5). Each virtual slice was obtained using the average Z projection of 6 to 10 consecutive reformatted slices using the Z-Project tool of the software Fiji, thus resulting into 9 to 30 μm thick reformatted slices (Figs. S4 and S5).

### Proteomic analysis: amelogenin-based sex estimation

Proteomic analyses through LC–MS/MS were conducted on the enamel of the left maxillary first molar to estimate the sex of AVH-1. Before sampling, laboratory-based X-ray microCT scan of the tooth was undertaken by use of a desktop Skyscan 1072 system (Bruker Corp., Kontich, Belgium). Scanning parameters were 50 kV and 197 µA for Voltage and current of the X-ray source, 1 mm aluminum filter, an exposure time of 5936 ms, image averaged on 2 frames, a total scan angle of 180° with an angular step of 0.9°. Reconstruction was obtained with NRecon software (Bruker Corp., Kontich, Belgium), with an isotropic voxel size of 10.42 µm, beam hardening correction (%) = 25, and ring artifact correction = 1.

Amelogenin-based sex estimation^56^enables the confident identification of the sex of an individual even when DNA seems compromised^22^. The analytical protocol is described in Lugli et al.^23^. A small fragment of the tooth (~ 10 mg; including both dentine and enamel) was sonicated with MilliQ water and quickly pre-cleaned with 5% HCl. Then, the specimen was digested through 250 μl of 5% HCl (suprapur grade). This solution was desalted and purified using a C_18_ silica SpinTip (Thermo Scientific) and dried down overnight under a class 100 laminar-flow hood in the clean room facility of the Department of Chemical and Geological Sciences (University of Modena and Reggio Emilia). Dry peptides were re-suspended using 35 μl of a water:acetonitrile:formic acid mixture (95:3:2) and analysed by LC–MS/MS (Dionex Ultimate 3000 UHPLC coupled to a high-resolution Q Exactive mass spectrometer; Thermo Scientific). A run time of 90 min was employed for the specimen and the blanks; see^23^ for details. The analysis of the AVH-1’s tooth was duplicated. To check the presence of amelogenin proteins we searched the ion chromatograms^22,23^, focusing on specific peptides as SM(ox)IRPPY (AMELY; [M + 2H]^+2^ 440.2233 m/z) and SIRPPYPSY (AMELX; [M + 2H]^+2^ 540.2796 m/z). Additionally, raw data were converted in Mascot generic format and searched against UniProt (constrained to *Homo sapiens*) and cRAP (contaminant database, 116 sequences). No proteolytic enzyme was selected in search parameters and one missed cleavage allowed. Deamidated asparagines/glutamine (NQ) and oxidated methionine (M) were set as variable modifications. Error tolerance was set at 10 ppm for the precursor ions and 0.05 Da for the product ions. False discovery rate was estimated through an automatic decoy database, with a probability threshold trimmed to a FDR < 1%. A specific protein was considered as identified if at least two significant peptides were observed.

### Ancient DNA extraction, library preparation, mitochondrial DNA capture and sequencing

After removing a thin layer of surface from the vertebral fragment of AVH-1 (plotted find #9237; Fig. S3), we drilled 9.5 mg and 8.1 mg of bone powder with a sterile dentistry drill. We produced 500 µl of lysate from each sample and used 150 µl aliquots for automated silica-based DNA extraction^57^, choosing binding buffer option “D”. Both DNA extracts were converted into single-stranded DNA libraries, which were subsequently quantified, amplified, and labelled with two unique indices^58^. Negative controls for the DNA extraction and the library preparation were carried along through all steps. The amplified libraries were pooled and sequenced on an Illumina HiSeq 2500 in paired end mode (2 × 75 cycles) with two index reads^59^. An aliquot of each library was further enriched for human mitochondrial DNA (mtDNA) in two successive rounds of hybridization capture^60–62^. Enriched libraries were pooled and sequenced on an Illumina MiSeq.

Paired-end sequence reads were overlap-merged into single-molecule sequences and adapters were trimmed using leeHom^63^. Merged sequences were mapped using the Burrows-Wheeler Aligner (BWA)^64^ with ancient DNA parameters (“–n 0.01 –o 2 –l 16500”)^65^, either to the modified human reference GRCh37 from the 1000 Genomes project (ftp://ftp.1000genomes.ebi.ac.uk/vol1/ftp/technical/reference/phase2_reference_assembly_sequence/) for the shotgun data, or to the revised Cambridge Reference Sequence (rCRS) for the mtDNA capture data. All downstream analyses were restricted to sequences with indices that perfectly matched the expected index combinations. We used bam-rmdup (version: 0.6.3; https://github.com/mpieva/biohazard-tools) to remove PCR duplicates and SAMtools (version: 1.3.1)^66^ to filter for fragments that were longer than 35 base pairs (bp) and had a mapping quality greater or equal to 25. Tables S2 and S4 summarize the number of DNA fragments retained after each step.

To evaluate if some of these fragments stem from authentic ancient DNA, we determined the frequency at which cytosines (C) are substituted by thymines (T) at their ends^67^. The elevated frequencies of C-to-T substitutions in the libraries of AVH-1 (Table S2 and S5) indicate the preservation of ancient DNA molecules in the specimen. Furthermore, these frequencies remained stable after filtering for fragments with a C-to-T substitution at the opposing end (‘conditional’ substitutions) (Tables S3 and S5), indicating that the majority of the data come from authentic ancient DNA^68^.

### Reconstruction of the mitochondrial genome and phylogenetic analysis

Using *schmutzi *^69^ (parameters: “—notusepredC —uselength”), we estimated 1% (95% confidence intervals (CI): 0–2.0%) of present-day human DNA contamination among all mtDNA fragments obtained from the AVH-1 libraries (Table S4). We next reconstructed the full mtDNA genome of AVH-1, first using all mapped fragments longer than 35 base pairs with a mapping quality of at least 25, and second using only fragments with a C-to-T difference to the reference genome at the first three and/or last three terminal positions. We called a consensus base at each position along the mtDNA genome that was covered by at least three DNA fragments and where at least 67% of fragments carried an identical base, as detailed in^68^. The reconstructed mitochondrial genomes from all fragments and from putatively deaminated fragments were identical, and identical to the consensus sequence called using *schmutzi.* We identified a haplogroup of AVH-1 to be U5b2b using HaploGrep2^70,71^ and PhyloTree database (PhyloTree.org, build 17)^72^.

A tip date for the AVH-1 mtDNA was estimated using the Bayesian method implemented in Beast2 (version 2.4.8)^73^. The reconstructed mitochondrial genome of AVH-1 was aligned to the mtDNA genomes of 54 present-day^74^ and 52 ancient modern humans of known radiocarbon age^62,75–82^, which served as calibration points for tip dating, using MAFFT v7.271^83^ (Table S6). The Vindija 33.16 Neandertal mtDNA genome^74^ was used as an outgroup. Using jModelTest2^84^ we determined the best-fitting substitution model for this dataset to be Tamura-Nei 93 with a fixed fraction of invariable sites and gamma distributed rates (TN93 + I + G). Models of rate variation and tree priors were investigated following^79,82^. We determined the strict clock model and Bayesian skyline to be the best fit to the data following a marginal likelihood estimation (MLE)^30^ analysis for model comparison and best support assessment. The resulting tree was visualized using FigTree (version: v1.4.2) (http://tree.bio.ed.ac.uk/software/figtree/).

### Sex determination from nuclear DNA

Sex of AVH-1 was determined from the shallow shotgun sequencing by counting the number of fragments which aligned to the X chromosome and the autosomes. The analysis was first performed using all mapped fragments longer than 35 base pairs with a mapping quality of at least 25, and then using only fragments with a C-to-T difference to the reference genome at the first three and/or last three terminal positions. Sex was determined based on the expected ratios of X to (X + autosomal) fragments for male and female individuals^85^.

### Analysis of the Burial Artifacts

#### Shell beads and pendants

Perforated shells were observed under ~ 20×–150× magnification using a DinoLite. Microscope images were used to take general measurements of the shells and their perforation, as well as to document the location of use-wear and ochre traces. A selection of 60 *Columbella rustica*, the single *Turritella* sp., and the four pendants were examined using a stereoscopic microscope (ZEISS AXIO Zoom V16) at the Diet and Ancient Technology (DANTE) Lab of La Sapienza, Rome. Author EC used the stereoscopic images to document the location of use-wear and ochre residues on each shell bead. The intensity of the use-wear was compiled for seven locations on each shell, which was used to calculate a use-wear score for each specimen.

The classification of the pendants’ taxonomy was performed using an experiment. Bivalve shells representing various taxa were purchased in Bordighera, Italy—including *Glycymeris pilosa*, *Spondylus gaederopus*, and *Arctica islandica*. An additional set of bivalves including *Ostrea* and *Halliotae* were obtained from Dr. Allen Desnoyer at Archaeology Southwest. The complete bivalves were broken into smaller fragments, placed into a Lorotone 3A rock tumbler with 1.5 lb of ceramic media and 2tbs of coarse grit, and tumbled for four days, followed by two days with 2tbs medium grit. Those fragments were observed under a DinoLite microscope to identify which taxa best approximate the burial ornaments. This analysis confirmed that *Glycymeris* fragments were used to make the pendants found in the burial. The pendants were also scanned using an Artec Space Spider scanner and these scans were processed in Artec’s Scan Studio 13 Professional to provide additional 3D visualization.

A preliminary experiment was performed to estimate the time required to perforate the *Glycymeris* pendants. Two fragments of tumbled *Glycymeris* were selected for their shape and size to match the specimens found in the burial. Those fragments were then drilled using lithic drills (knapped by Dr. Allen Desnoyer). The time required to create a usable perforation was recorded for each.

### Zooarchaeological, taphonomic and use-wear analysis of the bird of prey claw (PF# 6877)

The anatomic and taxonomic determinations were based on comparisons with the zoological collections of the Laboratory of Osteoarchaeology and Paleoanthropology (BONES Lab), in the Department of Cultural Heritage, University of Bologna (Ravenna).

Taphonomic analyses were carried out with the aid of Leica S9i stereomicroscope, Fujifilm, X-T3, blue light 3D surface scanner (Artec Space Spider).

Surface modification descriptive terms were derived from^86^. The incisions are striations with linear outlines of variable lengths, widths, and depths; they have a V-shaped section and display internal microstriation^87^. Fresh bone breakage on bird remains can be the result of several processes, such as disarticulation or the removal of fat and cartilage. These phenomena generate certain modifications (wrenching and peeling), which were grouped as over-extending damage. Peeling is defined as a roughened surface with parallel grooves and a fibrous texture^88^. Wrenching is the loss of cortical bone tissue related to disarticulation, affecting mainly the distal ends of humeri and radii and the articular ends of ulnae. In the case of a humerus, this process often generates a single hole at the distal end^89,90^.

Use-wear analysis was carried out by means of a Hirox KH 7700 3D digital microscope using two different optics: a MX-G 5040Z zoom lens equipped with an AD-5040 Lows objective lens (20–50×) and a coaxial vertical lighting MXG-10C zoom lens and an OL-140II objective lens (140–560×). Descriptive criteria (surface polishing, striations, rounding, faceting) for the functional interpretation of the claw were derived from the literature^91,92^.

### General faunal analysis

Faunal fragments from the burial pit were examined for taphonomic and taxonomic information. The identification of fragments to taxon was as specific as possible given most specimens were highly fragmented.Incidence lighting and a Dino-Lite Edge Microscope was used to analyze the surface of all faunal remains from the burial pit to identify tooth marks, cut marks, and percussion marks, as well as acid etching, trampling marks, and dendritic marks following published protocols^93–97^.

## Supplementary Information

Supplementary Information 1.

Supplementary Information 2.

Supplementary Information 3.

Supplementary Information 4.

Supplementary Information 5.

Supplementary Information 6.

Supplementary Video 1.

Supplementary Information 7.

Supplementary Information 8.

## Data Availability

All data are available upon request. Site data (e.g., 3D coordinates and photographs) are stored in databases managed by J.H., C.M.O, and C.G-M. Geoarchaeological samples are archived in a CouchDB database managed by C.E.M. (University of Tübingen). Microscope images of the perforated shells are archived, along with a FileMaker database holding measurements, use-wear, and ochre coverage in a Dropbox folder managed by C.G-M. Mass spectrometry proteomics raw data have been deposited to the ProteomeXchange Consortium via the PRIDE partner repository with identifier PXD017532. The mitochondrial genome sequence of AVH-1 is deposited in GenBank (accession number pending). Sequence reads from all libraries and corresponding negative controls are deposited at European Nucleotide Archive under study accession number PRJEB43051.
