# Supplementary material for: An infant burial from Arma Veirana in northwestern Italy provides insights into funerary practices and female personhood in early Mesolithic Europe
Source: Sci Rep. 2021 Dec 14;11:23735. doi: 10.1038/s41598-021-02804-z (PMC8671481; doi:10.1038/s41598-021-02804-z)
Supplement: Supplementary file 6 — Supplementary Information 6. [file 41598_2021_2804_MOESM6_ESM.pdf]

Supplementary Information

An infant burial from Arma Veirana in northwestern Italy provides insights into funerary practices and female personhood in early Mesolithic Europe

Jamie Hodgkins*#, Caley M. Orr#, Claudine Gravel-Miguel, Julien Riel-Salvatore, Christopher E. Miller , Luca Bondioli, Alessia Nava, Federico Lugli, Sahra Talamo, Mateja Hajdinjak, Emanuela Cristiani, Matteo Romandini, Dominique Meyer, Danylo Drohobytsky, Falko Kuester, Geneviève Pothier Bouchard, Michael Buckley, Lucia Mancini, Fabio Baruffaldi, Sara Silvestrini, Simona Arrighi, Hannah M Keller, Rocío Belén Griggs, Marco Peresani, David S. Strait, Stefano Benazzi, Fabio Negrino

*Correspondence to: jamie.hodgkins@ucdenver.edu

#These authors contributed equally to this work.

See article's main text for full author affiliations.

<u>The Supplementary Information includes</u>:

Supplementary Methods (pages 3 – 16)
Supplementary Results (pages 17 – 50)
Supplementary References 98 – 133 (pages 51 – 52)
Figs. S1 to S17 (see page numbers below)
Tables S1 to S15 (see page numbers below)
Caption for Movie S1 (page 19)
Legend for SuppDataFile1-ArmaVeiranaHumanRemains (page 29)
Legend for SuppDataFile2-ShellOrnamentData.xlsx (page 42)
Legends for 3D polygon models of burial artifacts (page 43)

Figure	*Page Number*		*Table*	*Page Number*
S1	4		S1	5
S2	7		S2	11
S3	8		S3	12
S4	10		S4	12
S5	10		S5	13
S6	18		S6	14
S7	19		S7	27
S8	22		S8	27
S9	23		S9	30
S10	25		S10	37
S11	28		S11	37
S12	39		S12	38
S13	41		S13	47
S14	44		S14	50
S15	45		S15	50
S16	48			
S17	49			

Other Supplementary Materials for this manuscript include the following as separate downloads:

Movie S1 (Arma Veirana fly through)
SuppDataFile1-ArmaVeiranaHumanRemains.xlsx
SuppDataFile2-ShellOrnamentData.xlsx
3D polygon models

- ArmaVeirana-PF3484-Glycymeris-pendant.ply
- ArmaVeirana-PF3854-Glycymeris-pendant.ply
- ArmaVeirana-PF3858-Glycymeris-pendant.ply
- ArmaVeirana-PF9228-Glycymeris-pendant.ply
- ArmaVeirana-PF6877-Bubo-talon.ply

SUPPLEMENTARY METHODS

Fieldwork at Arma Veirana and all sampling and analysis of the AVH-1 skeleton and other artifacts described here and in the main text was conducted under the auspices of Soprintendenza Archeologia, Belle Arti e Paesaggio per la città metropolitana di Genova e le province di Imperia, La Spezia e Savona via a permit formally issued to coauthor Fabio Negrino. All procedures followed the relevant requirements of the permitting authority.

Fieldwork and site documentation

Arma Veirana is mapped using a coordinate grid aligned to magnetic north and a virtual origin point near the center of the cave. Control points are marked on the walls to allow for triangulation of the total station's start position. These control points are provided in Fig. S1 and Table S1 to allow for future workers to reconstruct the excavation coordinate frame. Based on the grid, one-meter squares are named according to coordinates of their southwest corners (e.g., square 4N1E is four meters north and one meter east of the origin whereas 4N-1E is four meters north and one meter west). Each square is divided into 50 cm quadrants (ne, nw, se, sw) with excavation typically proceeding within a quad. Artifacts and specialist samples piece-plotted with the total station are assigned a unique identifying number (Plotted Find Number or Specialist Sample Number) using a barcode system(*98*). In addition, all sediments excavated from each quad have been sieved (down to 1.0 mm screen size) to recover small artifacts that were missed by excavators. Both piece-plotted and sieved artifacts are tagged with Lot Numbers in the Arma Veirana database, which localizes the material to quad and stratigraphic unit as recognized within that quad and established by natural geological boundaries (or 5-10 cm spits in cases of thick units).

All physical specimens are stored in museum boxes and sorted by the excavation level and archaeological unit (square) in which they were discovered. The materials are stored in the Laboratory of Paleontology at the University of Genoa. Micromorphology blocks and loose sediment samples for geoarchaeological processing have been transported to the Geoarchaeology laboratory at the Institute for Archaeological Sciences, University of Tübingen, Germany. The data pertaining to the 3D Coordinates of each specimen, taxonomic identification, lithic identification, sediment description, and photographs are stored in computer databases, with back-up copies existing in on-line data storage systems.

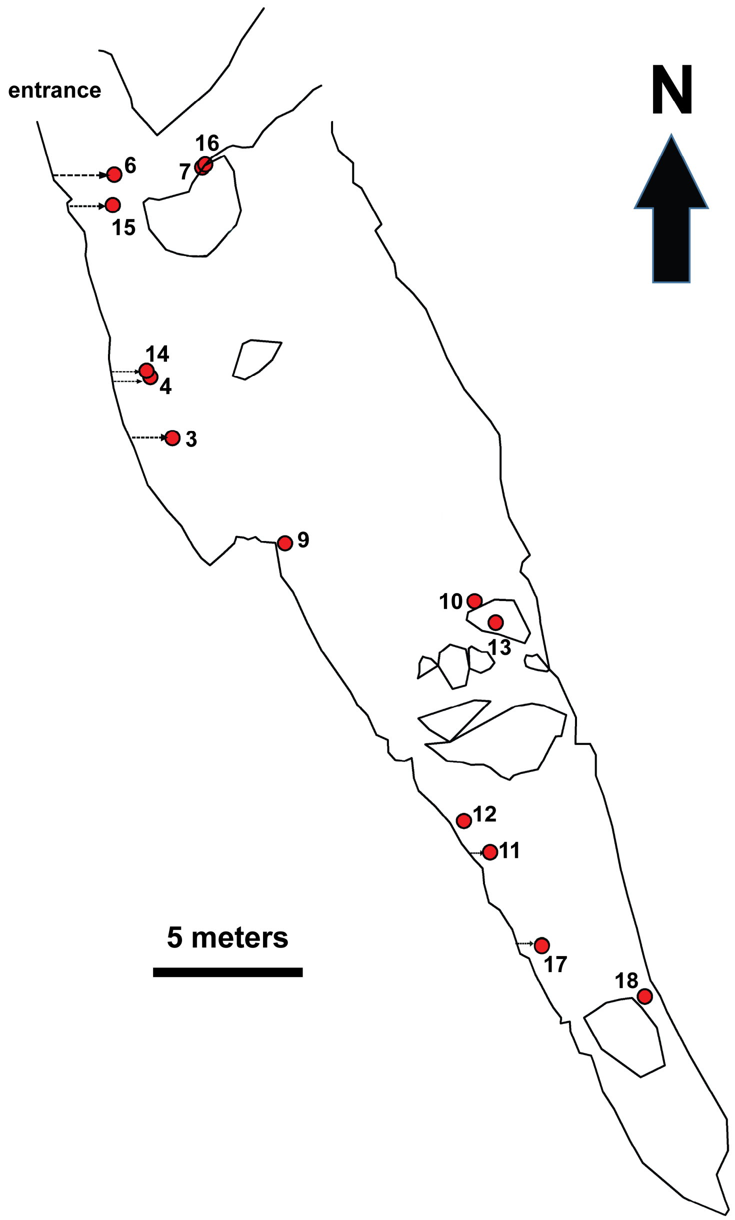

Fig. S1. Control points for triangulating 3D position within the Arma Veirana grid. These points can be used to determine the position of a total station allowing future excavations to maintain the same coordinate system. Lines emerging from the walls indicate a wall overhang. See Table S1 for the coordinates of each point. Control points 14, 15, and 16 are marked with metal bolts drilled into the rock wall (therefore will be the most long-lasting). Other control points are small depressions placed in the walls with a hand-held drill and dotted with a permanent marker. Note that control points 1, 2, 5, and 8 no longer exist. Map made using ArcGIS 10.5 (https://www.esri.com/).

Table S1. Control point coordinates.[*]

Point ID	Northing	Easting	Elevation	Location description
CP3	0.0095	-4.8736	1.8944	On western wall approximately 12 meters from the entrance just above head height.
CP4	2.0305	-5.6143	1.7362	On western wall approximately 10 meters from the entrance. Arm's length above head height.
CP6	8.7491	-6.8122	0.198	On western wall, approximately 4 meters from entrance and just above head height.
CP7	8.9822	-3.8834	-0.165	On medium sized boulder near entrance on top of the central rock shelf and 8cm below CP16.
CP9	-3.4813	-1.1207	2.102	On western wall, approximately 17 meters from entrance and just past the corner at chest height.
CP10	-5.402	5.1903	2.049	On large speleothem in center of cave against eastern wall at waist height.
CP11	-13.7089	5.7101	4.1579	On western wall ~26 m from entrance, behind central speleothem, at head height.
CP12	-12.6686	4.833	4.1706	On western wall ~25 m from entrance, behind central speleothem, at head height.
CP13	-6.1045	5.8985	3.2024	On northernmost speleothem in central cave against eastern wall on backside, head height.
CP14	2.2429	-5.7375	1.652	Center of metal bolt on western wall approximately 9 meters from the entrance. Arm's length above head height.
CP15	7.7378	-6.8618	0.2844	Center of metal bolt on western wall approximately 5 meters from the entrance. At head height.
CP16	9.0862	-3.7821	0.2405	Center of metal bolt on medium -sized boulder near entrance on top of central rock shelf.
CP17	-16.7983	7.4375	4.0002	On western wall in back of cave approximately 33 meters from entrance. Before rear speleothem, at chest height.
CP18	-18.496	10.8666	4.4813	On eastern wall in back of cave approximately 36 meters from entrance. Located on a flowstone column anterior to the rear speleothem at head height.

[*]Gray cells indicate control points on metal bolts implanted in stone walls of cave and are therefore the most durable. Other points exist only as depressions from a hand-held drill.

Photogrammetry

Progressive photogrammetric imaging (Fig. S2) was performed to document the 3D spatial relationships of the skeletal remains. As each artifact was exposed, it was photographed with marker chits whose coordinate positions were captured by a total station (Fig. S2B). These images were registered to progressively add each piece of the virtual burial.. During pose estimation, unique image dependent points and tie points, computed by matching keypoints between images are used. These points are increased in quantity and quality to provide better pose estimation, a crucial step to repetitive photogrammetry. A Sony a7 II was primarily used for photogrammetric imaging, the 42-megapixel sensor important to providing ample data to be used in pose estimation. A Zeiss 24-70mm and Sony 90mm macro lens performed the majority of photogrammetric imaging, with limited use from a Zeiss 16-35mm. Lenses were chosen to maximize spatial resolution while increasing depth of field, ensuring focus across the full observed burial region. Image clarity was heavily emphasized in a dense, generous acquisition strategy. To minimize image noise and scene shadows. external illumination with dual LED panels was required. Attention was paid to minimize shadows changing during imaging. Artifacts from AVH-1 burial were each imaged with 30-250 photos. Each model included at least four temporary chits whose coordinates were recorded via total station. Contextual models of the burial surrounding individual artifact, were created in the same way. While rough on-site model synthesis aided discussion, data was backed up daily, physically transported, and processed using Agisoft Photoscan on a cluster of 12 computer nodes. Nodes parallel processing capabilities varied with computational hardware, including Intel Xeon, Intel I7, AMD Ryzen CPUs, over 128GB RAM per, and Nvidia 1080Ti GPUs. Over 20,000 images were collected and processed, resulting in greater than 4 terabytes of associated data. Photoscan handled all photogrammetric processing, dense point clouds were computed at *Ultra High* and remained as point clouds; converseion into a mesh surface was avoided to maintain the highest level of geometric accuracy. A pierced shell ranged from 0.25 - 0.5 million points, with a 1.5 m^2 excavation context model containing 50-100 million points. These data were aggregated in the software Viscore (*99*) to produce a multiresolution digital surrogate of the site and AVH-1 burial. Viscore enabled the aggregation of aerial photogrammetry, cave photogrammetry, and burial artifacts and contexts. Visual parameters such as focal length can be modified and tools such as measurement, orthographic view, and applied digital raking light can be leveraged in the digital surrogate.

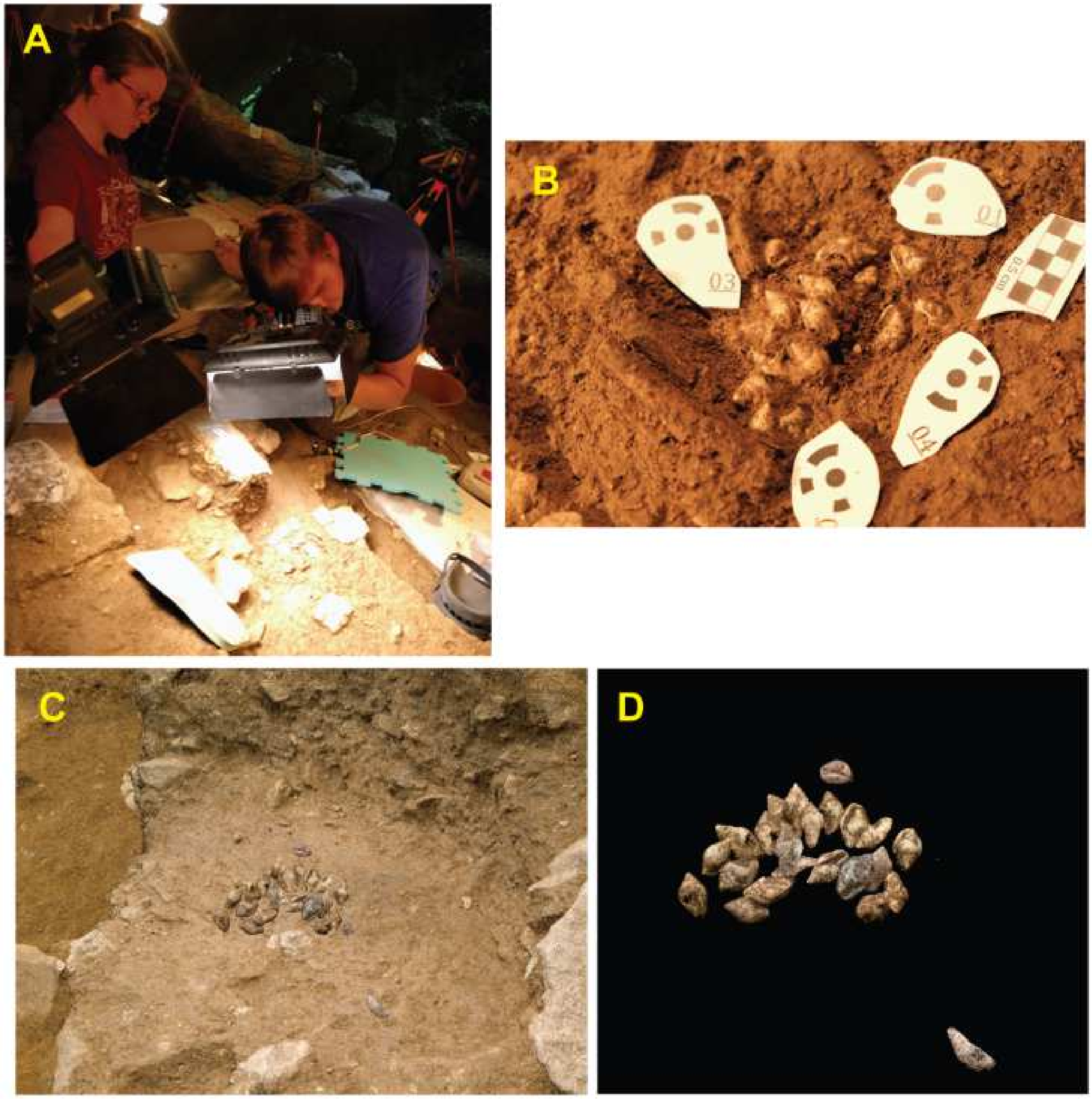

Fig. S2. Progressive photogrammetry of the burial excavation. A) Coauthors D.D. (with the camera) and G.P-B. (with light) taking photogrammetric images of the burial. B) Cluster of shell beads; each bead was individually photographed repeatedly and its position of each bead was recorded with a total station, and then removed to allow this procedure to be applied to deeper artifacts.. The photographs were registered to the coordinate system of the cave using the 3D positions of the target chits in the image. Subsequent photogrammetric sessions allowed for the merging of each piece of the burial to reconstruct the spatial position of each bone and artifact relative to one another even though each piece was removed separately. C) Reconstructed photogrammetric image of the *in situ* arrangement of pierced *Columbella rustica* beads in the inferred area of the pelvis with the modeled surroundings. D) Same cluster of *C. rustica* beads with the background removed digitally.

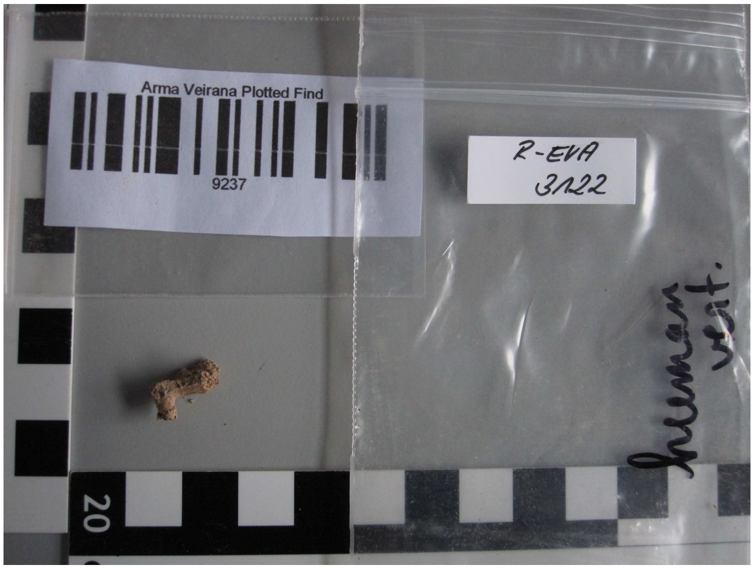

Fig. S3. Human vertebral arch (plotted find #9237) sampled for radiocarbon dating.

<u>Geoarchaeology: micromorphology</u>

Sediment blocks were collected in the field and secured with plaster-of-Paris bandages. The block samples were dried at 40˚C for over 24h and then indurated under vacuum with a mixture of unpromoted polyester resin and styrene (7:3 ratio) with methylethylketone peroxide (MEKP) used as a hardener (5ml per liter of resin/styrene mixture). Once the resin gelled, it was heated again at 40˚C for 24h to completely harden the sample. The blocks were then cut into slices using a rock saw and chips, measuring 6x9cm, were removed from the slices for thin sectioning. The thin sections were produced at the University of Tübingen by P. Kritikakis to a thickness of 30µm and were digitally documented using a flatbed (*100*) and high-resolution film scanner (*101*). The thin sections were examined and described under the naked eye and magnification using a petrographic microscope (Zeiss AxioImager) with plane polarized light (PPL), cross polarized light (XPL), and oblique incidence light (OIL) following procedures described by Courty et al. (*102*). Descriptive terminology follows that of Stoops (*103*).

Synchrotron scanning of the AVH-1 teeth

Measurements required for the virtual tooth histomorphometry were performed via propagation-based phase-contrast imaging using a filtered (filters: 1.5 mm Si + 1.0 mm Al) white X-ray beam with a mean energy of ca. 27 keV. For each tomographic scan 1800 sample radiographs were recorded during continuous rotation over a total scan angle of 180°. The detector used was a water-cooled, 16 bit, sCMOS macroscopic camera (Hamamatsu C11440-22C) with a 2048 × 2048 pixels chip coupled with a scintillator screen. The sample-to-detector distance was set at 200 mm. Samples were imaged with a multiscale approach setting the effective pixel size of the detector at $3.0 \times 3.0\ \mu m^2$ (named hereafter and in the main text, *medium spatial resolution*) and $0.9 \times 0.9\ \mu m^2$ (named hereafter and in the main text, *high spatial resolution*) yielding a maximum field of view of about $6.1 \times 4.1\ mm^2$ and 1.8x1.8 mm^2, respectively. A 45 µm-thick GGG:Eu (Gd3Ga5O12:Eu) and a 17 µm-thick GGG:Eu were respectively used for the two pixel settings with an exposure time/projection of 1.5 sec (3.0 µm pixel size) and 2.0 sec (0.9 µm pixel size). The axial slices were reconstructed using the filtered back-projection algorithm through the SYRMEP Tomo Project (STP, ver 1.2.5) software (*104*) developed at Elettra. The final volumes used to generate the virtual histological slices were created both without phase-retrieval pre-processing and applying the Paganin's single-distance phase-retrieval algorithm to the sample projections (*105*), setting the δ/β ratio to 20. After reconstruction with the phase-retrieval procedure, a sharpening filter was applied to the axial slices by the freeware Fiji (ImageJ ver 1.52s) (*106*). Virtual histological images are shown in the main text Figure 3A-B and supplemental figures S4 and S5.

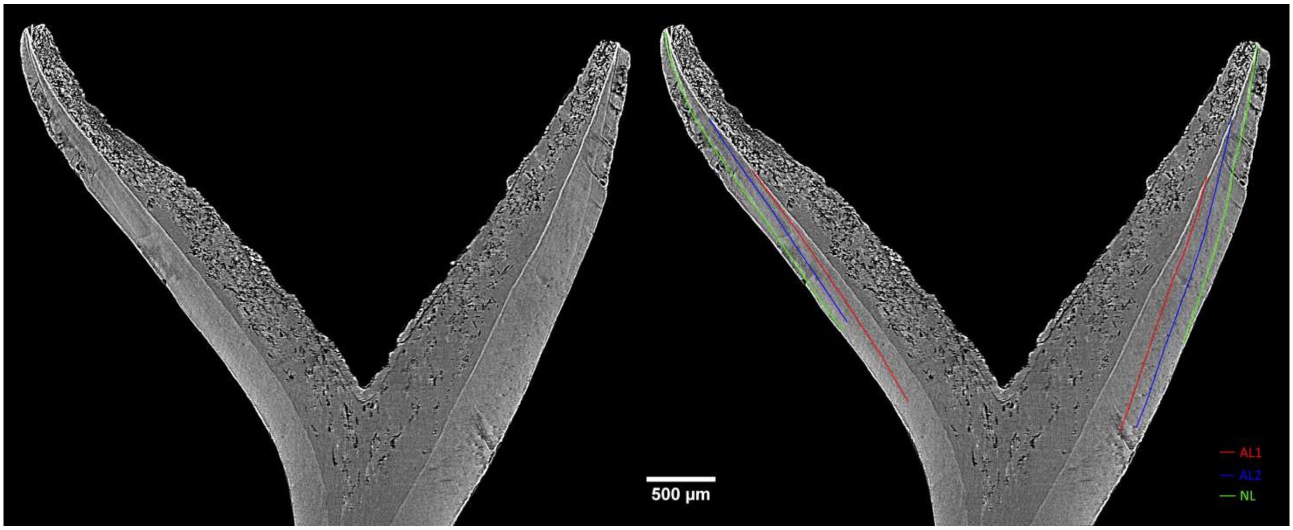

Fig. S4. Vestibular-lingual virtual histology section of the AVH-1c deciduous lateral incisor (synchrotron X-ray computed microtomography, pixel size 3 µm). Left: original reformatted section, 30 µm thickness. Right: reformatted section, 30 µm thickness with the NL and the ALs highlighted in color.

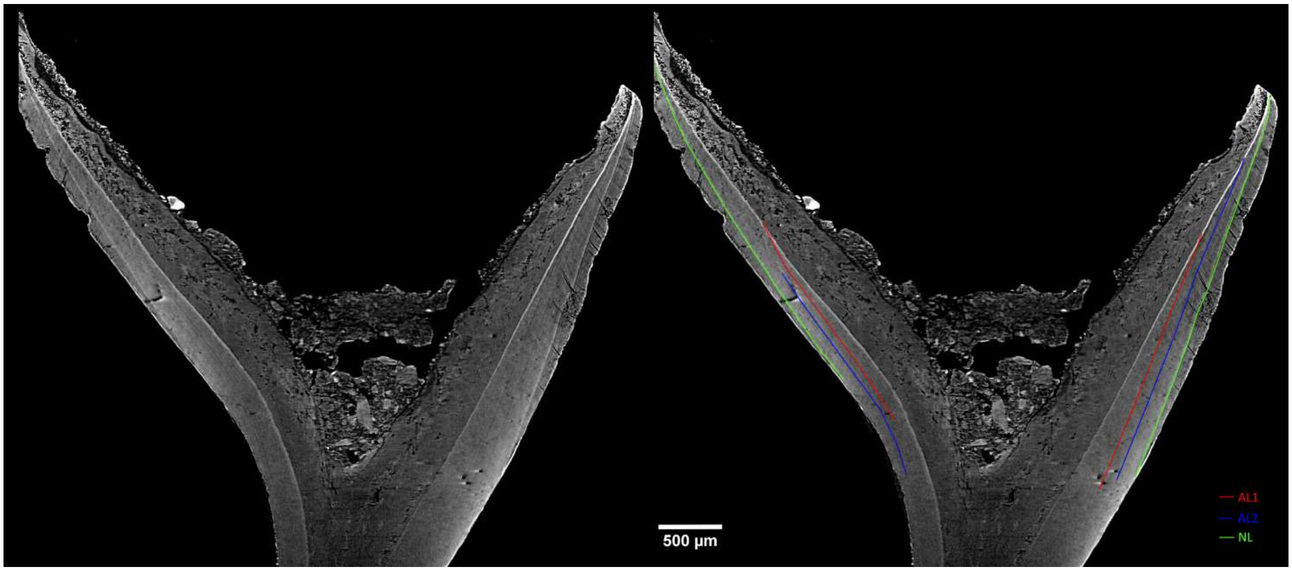

Fig. S5. Vestibular-lingual virtual histology section of the AVH-1d deciduous central incisor (synchrotron X-ray computed microtomography, pixel size 3 µm). Left: original reformatted section, 30 µm thickness. Right: reformatted section, 30 µm thickness with the NL and the ALs highlighted in color.

Ancient DNA extraction and analysis

Table S2 Overview of DNA extracts, libraries and shotgun sequencing summary. Fragments aligned to the modified human reference genome (GRCh37). Extraction (ENC) and library (LNC) negative controls are highlighted in gray.

Specimen	Amount of powder (mg)	Extract ID	Library ID	Number of molecules (qPCR)	Number of control oligonucleotide (qPCR)	Number of fragments generated	Number of mapped fragments ≥ 35bp, MQ≥25	Number of unique fragments ≥ 35bp, MQ≥25	Number of fragments with C→T substitutions ≥ 35bp, MQ≥25
Arma Veirana 1	9.5	E18495	A30952	3.50E+10	1.21E+06	6,663,417	22,224	22,104	6,658
	8.1	E18496	A30953	2.56E+10	1.20E+06	6,183,076	55,264	54,912	17,238
ENC		E18500	A30957	2.94E+08	1.15E+06	720,475	34	34	1
LNC			A30960	2.93E+08	1.31E+06	484,192	14	14	-

*ENC – extraction negative control; LNC – library negative control; qPCR – quantitative polymerase chain reaction; C – cytosine; T - thymine

Table S3 C-to-T substitution frequencies at the ends of nuclear DNA fragments. Fragments aligned to the modified human reference genome (GRCh37) of at least 35 bp and a mapping quality equal or greater than 25 were used for calculating the substitution frequencies. 95% binomial confidence intervals (CI) are provided in brackets. Extraction (ENC) and library (LNC) negative controls are highlighted in gray.

		All fragments		Fragments with C-to-T substitutions at opposing end	
**Specimen**	**Library ID**	**5' C-to-T (%) [95% CI]**	**3' C-to-T (%) [95% CI]**	**5' C-to-T (%) [95% CI]**	**3' C-to-T (%) [95% CI]**
Arma Veirana 1	A30952	50.2 [48.7-51.6]	38.4 [36.9-39.8]	48.4 [43.2-53.6]	38.5 [34.1-43.1]
	A30953	50.2 [49.3-51.1]	38.3 [37.4-39.2]	45.4 [42.3-48.6]	36.5 [33.7-39.2]
	merged libraries	50.2 [49.4-50.9]	38.3 [37.5-39.1]	46.3 [43.6-49.0]	37.0 [34.7-39.4]
ENC	A30957	0 [0.0-45.9]	0 [0.0-84.2]	NA [NA]	NA [NA]
LNC	A30960	0 [0.0-60.2]	0 [0.0-97.5]	NA [NA]	NA [NA]

*ENC – extraction negative control; LNC – library negative control; CI – confidence interval; C – cytosine; T – thymine

Table S4 General characteristics of libraries enriched for human mitochondrial DNA (mtDNA). Fragments mapped to the revised Cambridge reference sequence with a mapping quality of at least 25 and longer than 35 bp were utilized for calculating the average mtDNA coverage. Extraction (ENC) and library (LNC) negative controls are highlighted in grey.

**Specimen**	**Library ID**	**Number of fragments generated**	**Number of mapped mtDNA fragments ≥ 35bp, MQ≥25**	**Number of unique mtDNA fragments ≥ 35bp, MQ≥25**	**Coverage fragments ≥ 35bp, MQ≥25**	**Number of fragments with C→T substitution ≥ 35bp, MQ≥25**	**Coverage fragments with C→T substitution ≥ 35bp, MQ≥25**	**Contamination estimate *schmutzi* % (95% CI)**
Arma Veirana 1	A30952	746,503	303,178	136,791	463.57	38,547	127.55	1 (0-2)
	A30953	322,109	122,499	75,354	258.16	21,277	71.68	1 (0-2)
	merged libraries	1,068,612	425,677	212,145	721.73	59,824	199.23	1 (0-2)
ENC	A30957	9,413	179	35	0.13	1	0	
LNC	A30960	6,529	147	36	0.14	1	0	

*ENC – extraction negative control; LNC – library negative control; qPCR – quantitative polymerase chain reaction; C – cytosine; T - thymine

Table S5 C-to-T substitution frequencies at the ends of mtDNA fragments. Fragments mapped to the revised Cambridge reference sequence with a mapping quality of at least 25 and longer than 35 bp were used for calculating the substitution frequencies. 95% binomial confidence intervals (CI) are provided in brackets. Extraction (ENC) and library (LNC) negative controls are highlighted in grey.

		All fragments		Fragments with C-to-T substitutions at opposing end	
Specimen	Library ID	5' C-to-T (%) [95% CI]	3' C-to-T (%) [95% CI]	5' C-to-T (%) [95% CI]	3' C-to-T (%) [95% CI]
Arma Veirana 1	A30952	45.4 [44.9-46.0]	34.7 [34.2-35.2]	44 [42.2-45.9]	34.6 [33.0-36.2]
	A30953	44.8 [44.1-45.5]	34.4 [33.6-35.1]	46.5 [44.0-48.9]	34.8 [32.8-36.9]
	merged libraries	45.2 [44.8-45.6]	34.6 [34.1-35.0]	44.9 [43.4-46.4]	34.7 [33.4-35.9]
ENC	A30957	0 [0.0-41.0]	0 [0.0-23.2]	NA [NA]	NA [NA]
LNC	A30960	0 [0.0-36.9]	0 [0.0-41.0]	NA [NA]	NA [NA]

*ENC – extraction negative control; LNC – library negative control; CI – confidence interval; C – cytosine; T – thymine

Table S6 Ancient modern human mtDNA genomes and their radiocarbon dates used for calculating Arma Veirana 1 tip date in Beast2 (*73*). All radiocarbon dates were calibrated using IntCal20 (*107*) and OxCal 4.4.2 (*108*).

Individual	Radiocarbon date ID	Raw radiocarbon date	Date type	Publication of the date	Cal BP 95.4%
Bacho Kiro CC7-335	ETH-86772	42,450±510	Direct-UF	Hublin et al, 2020	45,930-44,420
Bacho Kiro BB7-240	ETH-86770	41,850±480	Direct-UF	Hublin et al, 2020	45,550-43,940
Ust'Ishim	OxA-25516 & 30190	41,400±950	Direct-UF	Fu et al, 2014	45,930-42,900
Bacho Kiro CC7-2289	ETH-86771	40,600±420	Direct-UF	Hublin et al, 2020	44,400-42,990
Bacho Kiro AA7-738 (same as F6-620)	ETH-86769	39,750±380	Direct-UF	Hublin et al, 2020	43,930-42,580
Oase1	GrA-22810 and OxA-11711	34,950±900	Direct-UF	Trinkaus et al, 2004	41,860-37,950
Tianyuan	BA-03222	34,430±510	?	Fu et al, 2013	40,850-38,070
Kostenki14	OxA-X-2395-15	33,250±500	Direct	Marom et al, 2012	39,380-36,670
Goyet Q116-1	GrA-46175	30,880±170	Direct-noUF	Posth et al, 2016	35,630-34,720
Bacho Kiro BK-1653	ETH-86768, AIX-12024	30,570±120	Direct-UF	Hublin et al, 2020	35,290-34,610
Cioclovina1	OxA-15527	28,510±170	Direct-UF	Soficaru et al, 2007	33,300-32,010
Vestonice16	Aix12030	27,220±110	Direct-UF	Fewlas et al, 2019	31,530-31,080
Vestonice43	Aix12032	27,070±110	Direct-UF	Fewlas et al, 2019	31,280-31,030
Vestonice13	Aix12027	27,040±100	Direct-UF	Fewlas et al, 2019	31,240-31,030
Vestonice14	Aix12028	26,760±100	Direct-UF	Fewlas et al, 2019	31,150-30,860
BerryAuBac1	SacA-5455	6,325±35	Direct	Posth et al, 2016	7,320-7,160
Bockstein	UtC-7887; UtC-6796	7,350±70 and 7,460±60	layer date	Benazzi et al, 2011	8,370-8,030

Brillenhohle	OxA11054; OxA23414	12,470±65 and 12,535±50	Direct	Simon, 1993	15,060-14,330
CuiryLesChaudardes1	GrA-28268	7,400±60	Direct	Posth et al, 2016	8,350-8,030
Falkenstein	ETH-7615	8,185±80	Direct	Trinkaus, Svoboda, 2006	9,430-8,990
Felsdach	B-933	7,770 ± 120	layer date	Bramanti et al, 2009	8,990-8,370
Goyet2878-21	GrA-62455	22,360±110	Direct	Posth et al, 2016	27,010-26,370
GoyetQ-2	GrA-46168	12,650±50	Direct	Posth et al, 2016	15,260-14,920
GoyetQ376-19	GrA-54026	23,260 +110, -100	Direct	Posth et al, 2016	27,720-27,300
GoyetQ376-3	GrA-60034	29,370 +180, -170	Direct	Posth et al, 2016	34,350-33,450
GoyetQ53-1	GrA-46169	23,920±100	Direct	Posth et al, 2016	28,390-27,770
GoyetQ55-2	GrA-54031	23,270 +120, -110	Direct	Posth et al, 2016	27,730-27,290
GoyetQ56-16	GrA-5999	22,100±100	layer date	Posth et al, 2016	26,800-25,970
HohleFels10	H5312-4907 and H5119-4601	12,770±220 and 13,085±95	layer date	Housley et al, 1997	15,940-15,010
HohleFels49	H5312-4907 and H5119-4601	12,770±220 and 13,085±95	layer date	Housley et al, 1997	15,940-15,010
HohleFels79	MAMS-25564	12,490±70	Direct	Posth et al, 2016	15,050-14,300
HohlensteinStadel	ETH-5732	7,835±80	Direct	Orschiedt, 1999	8,990-8,420
Iboussieres25-1	GrA-43700	10,140±50	layer date	Posth et al, 2016	11,950-11,400
Iboussieres31-2	GrA-43700	10,140±50	layer date	Posth et al, 2016	11,950-11,400
Iboussieres39	GrA-43700	10,140±50	Direct	Posth et al, 2016	11,950-11,400
LaRochette	OxA11053 and OxA23413	23,630±130 and 23,400±110	Direct	Benazzi et al, 2011	29,000-26,910

LesCloseaux3	OxA-7109(Ly-612)	8,870±130	Direct	Valentin et al, 2008	10,240-9,550
MareuilLesMeaux1	GrN-27225	8,320±90	Direct	Valentin et al, 2008	9,530-9,020
Ofnet	OxA1574	7,480±80	layer date	Orschiedt, 1999	8,430-8,040
Paglicci108	F-52	23,470±370	layer date	Azzi et al, 1974	28,600-27,150
Paglicci133	UTC-1414 and UtC-1789	28,100±400 and 29,300±600	layer date	Palma di Cesnola (2004)	34,230-31,770
Paglicci71	F-66	15,460±220	layer date	Azzi et al, 1977	19,200-18,250
Ranchot88	GrA-38019	8,985±40	Direct	Posth et al, 2016	10,240-9,910
Rigney1	Ly-6515 (OxA)	12,930 ± 55	Direct	Cuppilard et al, 2015	15,650-15,270
Rochedane	GrA-41739	11,120±50	Direct	Cuppilard et al, 2015	13,160-12,900
Boshan	MAMS-13530	7,368+-34	Direct	Fu et al, 2013	8,320-8,030
Loschbour	OxA-7338	7,205+-50	Direct-UF	Lazaridis et al, 2014	8,170-7,930
Iceman	Oxa37371-6 and OxA-3419-21	4,540+-55	Direct	Ermini et al, 2008	5,450-4,970
Eskimo Saqqaq	OxA20656	4,044 ± 31	Direct	Gilbert et al, 2008	4,790-4,410
Oberkassel	OxA-4792	11,570+-100	Direct	Fu et al, 2013	13,730-13,180

SUPPLEMENTARY RESULTS

Arma Veirana: Excavation History, Site Information, and Geological Context of the Burial

Excavations at Arma Veirana cave from 2015–2018 were led by authors (and co-project-directors) Benazzi, Hodgkins, Miller, Negrino, Orr, Peresani, Riel-Salvatore, and Strait. Critical field expertise and leadership was also contributed by postdoctoral scholar Gravel-Miguel and PhD student Pothier Bouchard with 3D imaging directed by Meyer and his team. This campaign constituted the first formal systematic investigation of Arma Veirana, which was identified as a Paleolithic site by Giuseppe Vicino, who conducted preliminary studies of artifacts recovered from looter pits in the cave.

In addition to the fly-through in Movie S1, an interactive 3D photogrammetric model of Arma Veirana is available to view online (https://bit.ly/3jCq4zC). The cave is 11m wide at its greatest width and 44 m long, tapering to a point at the back. From the northern entrance of the cave, the long axis is oriented to the southwest. The vaulted ceilings form an inverted V that reaches a maximum height of approximately 10 m from the modern floor. The modern cave floor slants downward from the posterior of the cave. Excavations near the toe of the slope have revealed rich Middle Paleolithic deposits that are (to date) known from four distinct layers [stratigraphic aggregates Black Mousterian (lower-most excavated), Granular, Consolidated Strong Brown, and Rocky Brown (uppermost)] and topped by likely sterile sediments separating them from deposits of terminal Pleistocene and early Holocene age.

The Mousterian-bearing deposits are primarily revealed in an east-west trench spanning squares 5N-3E to 5N1E (Figs. S6 and S7) and connecting two looter pits (located in the west half of 5N-1E and east half of 5N1E). This primary trench was designed to investigate the general stratigraphy and were the focus of the 2015 and 2016 field seasons. In 2017, two north-south trenches (along the -1E and 1E lines) were extended in the cave with the intention of intersecting later intact cultural horizons. Such deposits were hypothesized to exist upslope based on artifacts recovered from disturbed sediments deposited by an erosional gulley near the midline of the cave that had cut into the uppermost Mousterian-bearing layer (Rocky Brown). Figure S7 shows a general view of these excavations. Excavation southward in superficial deposits to the east of the erosional gulley (quads 3N1E ne and 2N1E ne) recovered six pierced shell beads and scattered lithics. Being close to the surface, these deposits were likely disturbed and not in original context but suggested the erosion of an artifact bearing stratum to the south. Human infant hand bones and deciduous teeth were then recovered from sediments sieved from quad 2N1E ne, suggesting a possible inhumation. Following this, the articulated line of shell beads and pendants associated with a human long bone (right humerus) and likely cranial fragment were excavated within the west wall of the quadrant bringing further proof that we were in the presence of a partially intact burial with the top of the remains appearing approximately 10 cm below the modern surface. In fall 2017, proteomic analysis by Zooarchaeology by Mass Spectrometry (ZooMS) (*109*) conducted by G.P-B. and M.B. at the University of Manchester on a small flake of the cranial fragment confirmed that the bones were human (see results in the "Skeletal remains" section below). Full excavation of the rest of the remains commenced in the 2018 field season beginning 8 July with completion on 31 July.

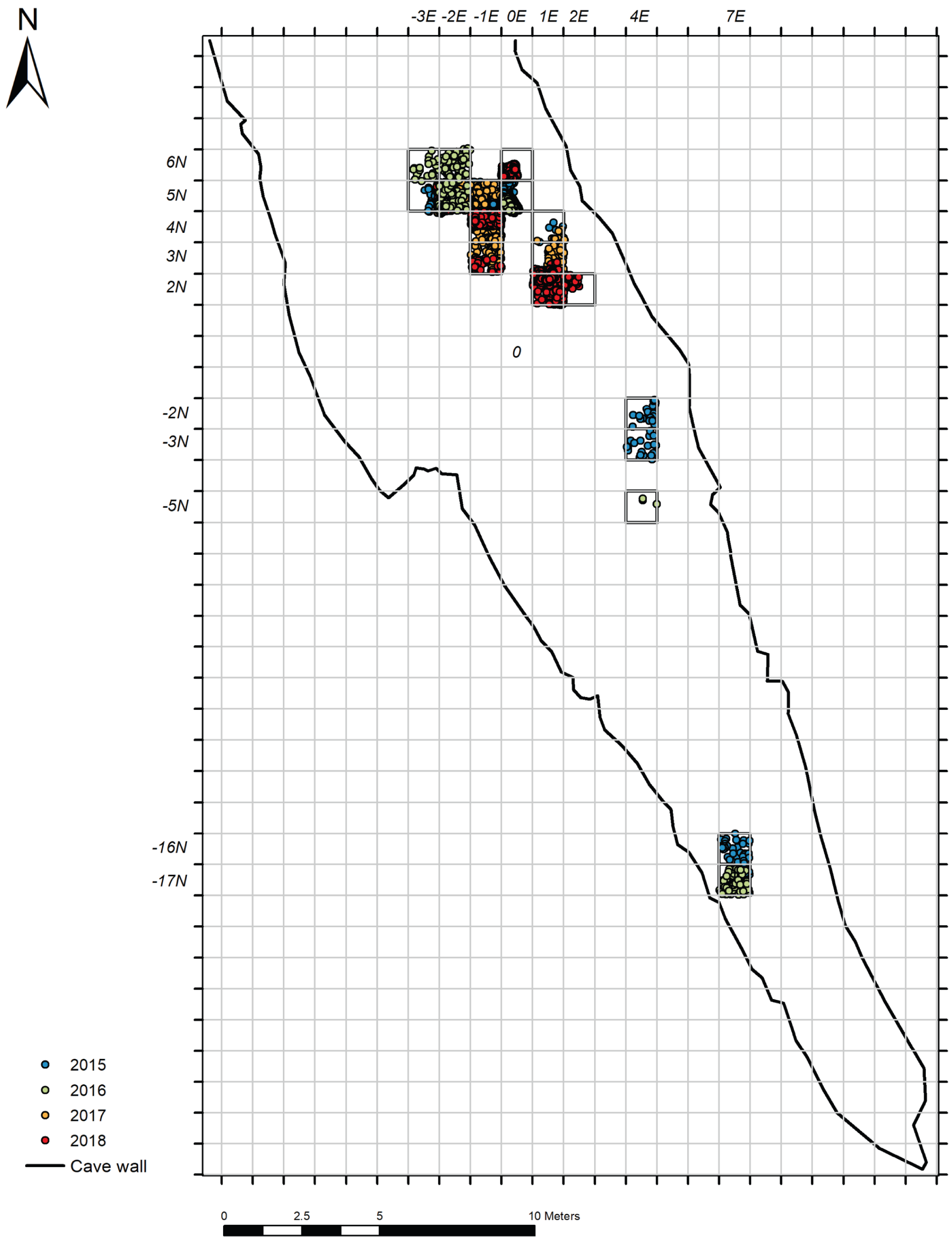

Fig S6. Plan-view map of the Arma Veirana excavations carried out between 2015-2018. Color-coded dots indicate plotted finds. The cave entrance is at the north end. The primary excavation has occurred at the north end of the cave and the smaller trenches to the south (starting with -2N4E) were early test pits. The AVH-1 burial was recovered primarily from the 2N1E square (mostly within its northeast quad). Shell beads likely associated with the burial and apparently eroded out were recovered from sieved sediments in 3N1E just preceding the first discovery in 2017 of the *in situ* remains. Map made using ArcGIS 10.5 (https://www.esri.com/).

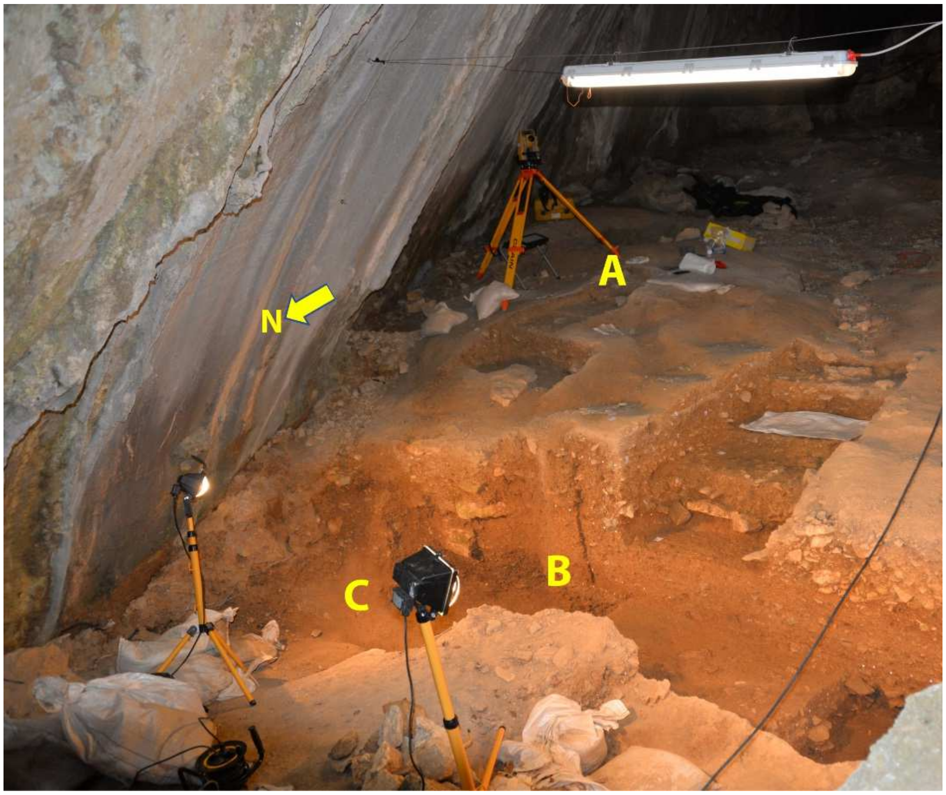

Fig. S7. View of Arma Veirana excavations from near the entrance at northern end of the cave looking to the southeast (July 2018). (A) marks the burial from square; (B) marks the lower trench exposing Mousterian-bearing deposits (for reference, the letter B is in square 5N0E). (C) marks the position of a large looter pit located at the far eastern end of the lower trench that had been dug prior to the beginning of formal excavations in 2015.

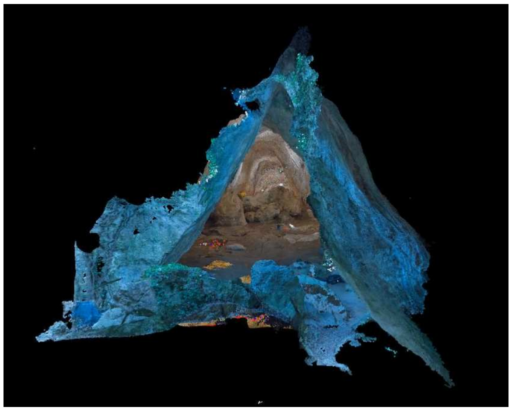

Movie S1. Arma Veirana fly through (available for download as a .mp4 file in the Supplementary Information section in the online journal). The video begins at the entrance, which is located at the northern end of the cave. It pauses at the burial location and then continues to the back of the cave. Movie and 3D model on which is based were generated by coauthors DM, DD, and FK.

General geological and stratigraphic situation of Arma Veirana

The modern-day surface in Arma Veirana has been subjected to significant erosional action that has removed Pleistocene- and early-Holocene-aged deposits from the cave (Fig. S8). It is difficult to pin-point exactly when erosion began or what caused the cave to shift from net accumulation of sediment to net loss. The effects of erosion are not uniform across the cave surface but are most pronounced towards the entrance of the cave where Mousterian-aged deposits occur almost directly at the modern surface. Toward the back of the cave, younger deposits are preserved and exposed. The modern surface of the cave exhibits a dendritic pattern of rills with the main gully running through the center of the cave, so that between the rills, the flanks of the cavity contain small remnant mounds of intact deposits, many of which appear buttressed and protected from erosion by large pockets of boulders and rockfall. The burial pit was encountered in one of these remnant mounds, to the east of the main erosional rill, in 2N1E.

Excavations at Arma Veirana have focused on several locations within the cave, exposing stratigraphic sections that span several lithological units tagged as or stratigraphic aggregates (StratAggs) in our excavation system (equivalent to what are often called "layers"). Since the excavations have occurred in laterally distinct localities, and direct correlation across the site is not always confirmable, we decided to give the StratAggs abbreviated names rather than numbers, to avoid confusion in the future, when direct correlation will likely be possible (Fig. S9). The lowest StratAggs reached during excavation are designated BM0 and BM3 ("Black Mousterian") and were first exposed in squares 5N1 0E and 5N1 3E. The subscripts indicate the squares in which the StratAggs were first identified (BM0 in 5N1 0E and BM3 in 5N1 3E). Similarity in lithology and elevation strongly suggests that these StratAggs are the same lithological unit; however, this remains to be conclusively demonstrated through further excavation.

Both BM3 and BM0 are notable for their dark color, with BM0 appearing brown (10YR 3/2) and BM3 appearing dark yellowish brown (10YR 3/4). The dark coloration of these StratAggs is emphasized by the inclusion of numerous sand- and gravel-sized fragments of charcoal and burnt bone, as well as manganese oxide staining. Micromorphological analysis of these units demonstrate a high proportion of finely comminuted charcoal and organic matter, giving it a dark color in the field. Both BM3 and BM0 are sandy silts, with an apparent higher sand content in BM3 and both exhibit a crumb structure. Éboulis is present, although less than in some overlying units, and is mostly sub-angular with blocks on average 10cm in size, displaying a horizontal orientation.

BM3 and BM0 are both overlain by G (granular) with a generally clear contact, which appears clearer over BM3 where it is marked by a thin lens of charcoal. G is a medium sandy silt with granules and gravel, with color varying around 10YR 4/4 to 10YR 4/3 (brown to dark yellowish brown). It exhibits a coarse crumb structure. It has a relatively high proportion of éboulis, which is mostly dominated by sub-angular to sub-rounded clasts that appear weathered and are on average 5-10cm in size. The proportion of éboulis decreases to the east, however, where éboulis is more rare. Portions of G in square 5N1 0E appear cemented by secondary carbonate, forming a weak breccia.

G (granular) is overlain by CSB (compact strong brown) with a generally diffuse but clear contact, which locally dips to the north. It is a clayey silt with fine sand and gravel and appears more compact and displays a massive structure. The color is dark yellowish brown (10YR 4/4). Larger blocks of éboulis are relatively rare and consist mostly of 5cm-sized sub-angular to angular clasts which are locally organized into horizontally oriented lenses.

Rocky Brown (RB) is locally truncated by the erosional surface and rills but it is overlain in places by modern surficial deposits, (StratAggs "S" and "D") which are expressed either as sedimentary infillings within the rill system or as alteration surfaces formed directly on RB. Toward the back of the cave (south), where erosion has removed less of the intact deposits, RB is directly overlain by Yellow Silt (StratAgg YS). RB is a clayey silt with fine sand and gravel and displays a weakly developed subangular blocks structure; the color is dark yellowish brown (10YR 4/4). RB contains a relatively high proportion of subangular to angular blocks of éboulis which are generally 10-15cm in size. These occur in higher proportion than in CSB. They exhibit an orientation fabric that trends towards the north, with a slight dip to the northeast.

The contact between YS and RB is most obvious in profiles exposed in square 4N 1E, where it is diffuse and marked by a clear decrease in the abundance of the coarse fraction (gravel and larger) as well as a more compact, massive structure. The color of YS here is similar to RB (10YR 4/4) although elsewhere, particularly in 2N1E, it appears more yellowish in color. In the field, YS appears to be a clayey silt with minor sand and gravel components. Larger blocks of éboulis are rare, with most occurring between 5-7cm in size. They appear subrounded and display no preferred orientation.

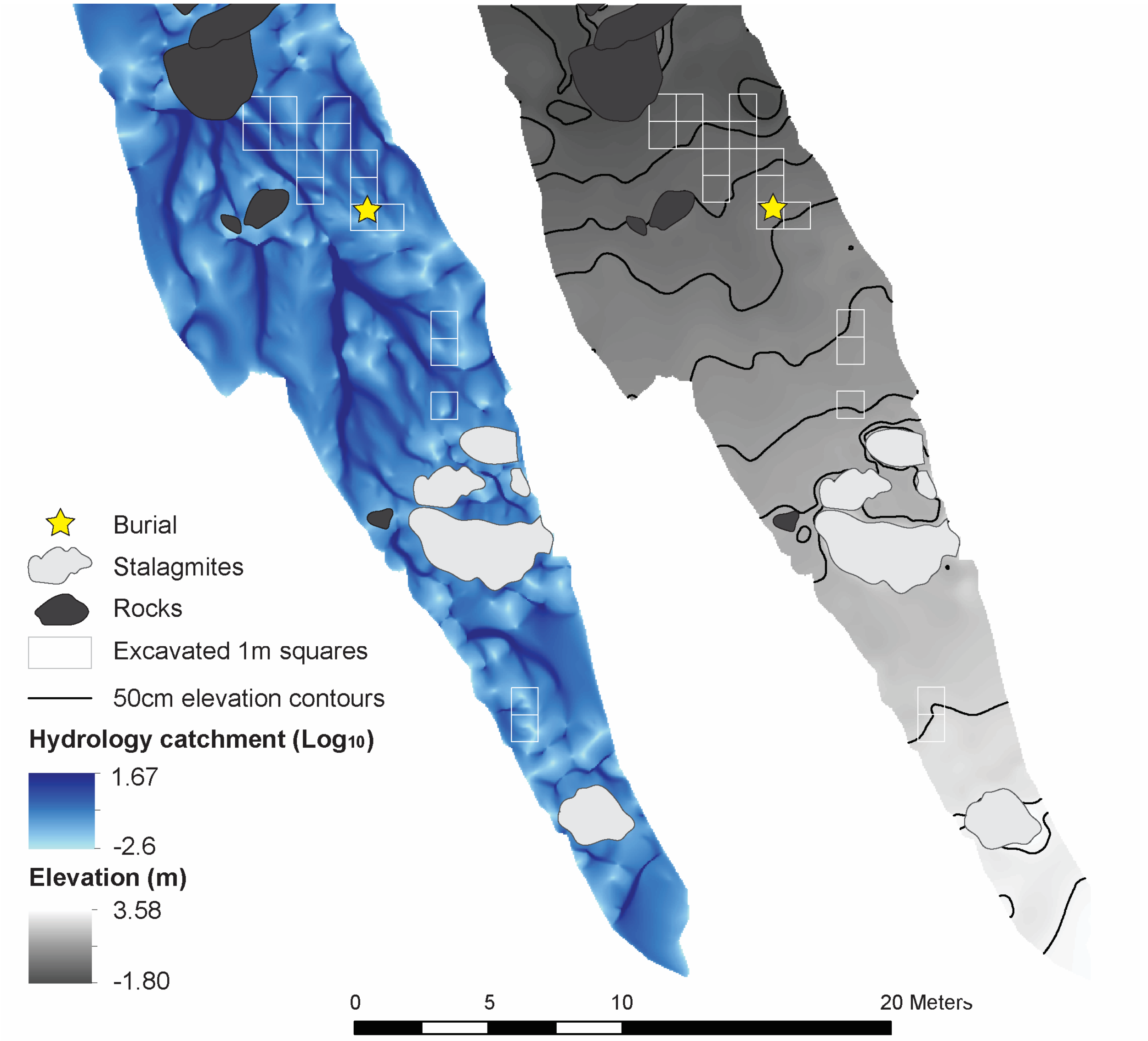

Fig. S8. A digital elevation model of the cave surface indicating the location of large boulders exposed on the surface and a model of the rill system which developed on the cave floor surface. Erosion impacted Pleistocene and early Holocene deposits and is most extreme toward the entrance of the cave. Periodic flowing water has exacerbated the erosion, forming a rill system within the cave. Erosion related to flowing water is largely concentrated along the central axis of the cave, leaving remnants of preserved sediment along the flanks including where the infant burial was located. The hydrology catchment and elevation models were built based on mapping of the cave floor using total stations.

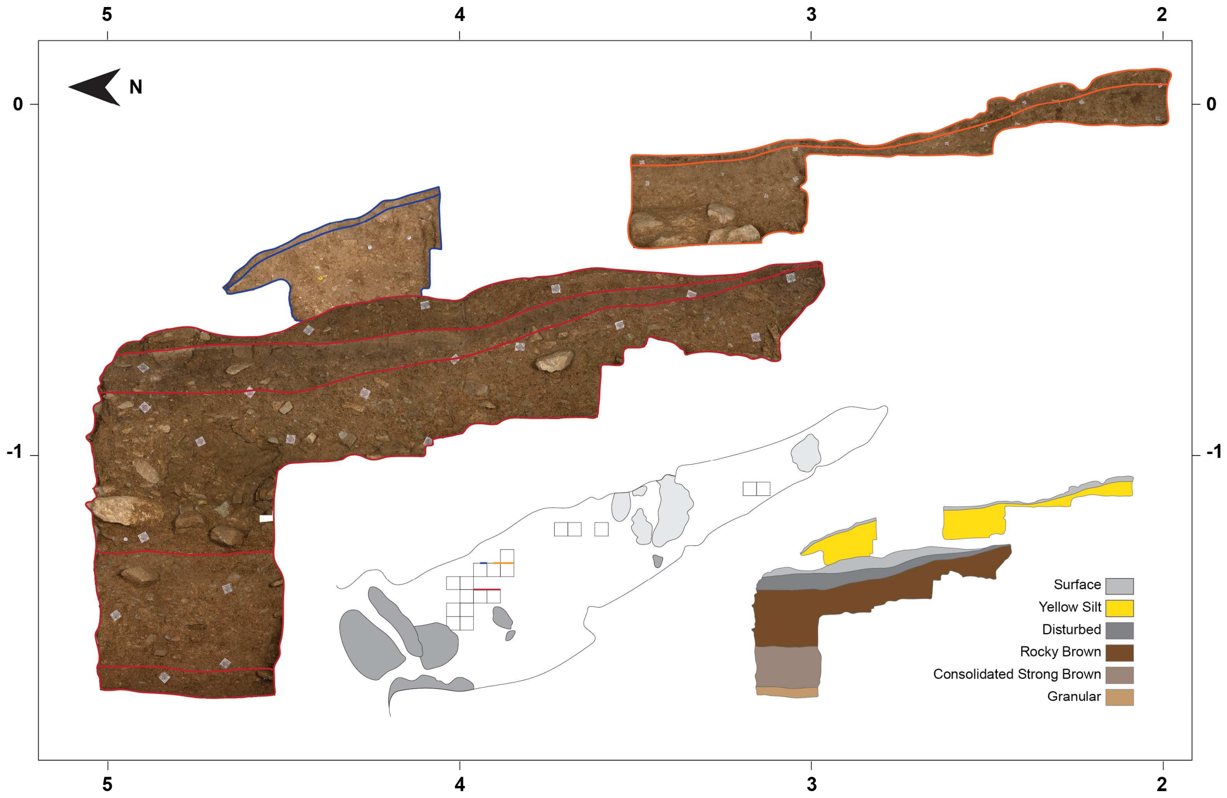

Fig. S9. Photograph and stratigraphic drawing of N-S profile. The horizontal axis of the outside box represents the north-south axis of the cave's coordinate system, while the vertical axis represents depth (both in meters). The composite image here is a projection of two profiles. The more western profile (in squares 3N-1E and 4N-1E; refer to Fig. S6 to view the square numbers) is located closer to an erosional rill, and therefore does not contain the layer YS, which is only exposed in excavations along the flank of the cave as represented in the more eastern profile (in squares 2N1E, 3N1E, and 4N1E). Excavations have exposed deposits below Granular (BM0 and BM3), but they have not been reached yet in these excavation units.

Description of the burial feature and associated deposits

In 2N1E, the feature containing the infant remains was located directly at the modern surface and appears as an intrusive pit into YS (yellow silt). The pit infilling was readily distinguishable from the surrounding YS sediment through its higher proportion of coarse material, including obvious anthropogenic components such as charcoal and bone. These components give the pit infilling a much darker color than the surrounding YS sediment. The outline of the pit was more readily distinguished to the south, where it is significantly deeper; to the north the pit appears to have been truncated and partially eroded. We collected micromorphological block samples to investigate the nature of the pit infilling and the surrounding YS sediment. Sample AV-18-01 was collected from the pit filling directly above the cranium and AV-18-02 includes YS outside of the pit feature. Below we provide micromorphological descriptions of the deposits and associated images (Fig. S10).

Yellow Silt

The sample including YS contains ca. 15% medium gravel, consisting largely of sub-rounded fragments of marble, and fine gravel-sized, subangular to subrounded fragments of sandstone/quartzite, shale/schist, speleothems and tufa. Fine gravel to coarse sand-sized fragments of bone, including some that are indurated with secondary carbonate are also present within the coarse fraction. Individual, sand-sized grains of quartz are present and likely derive from the breakdown of bedrock within the cave. Gravel-sized fragments of gastropod shells are present. Anthropogenic input appears minimal to absent, although a single gravel-sized fragment of charcoal is present. The coarse fraction (sand-sized and larger) displays a random distribution pattern. The micromass is a clayey silt, with silt-sized grains of mica present. Overall the sample displays a porphyric c/f-related distribution with crystallitic b-fabric indicating a high proportion of calcium carbonate within the micromass. The voids are mostly channels and vughs, with some planar voids defining a weakly developed subangular blocks microstructure. Pedofeatures include localized iron hypocoatings and calcium carbonate hypocoatings. Coarse sand-sized fragments of laminated, dusty clay coatings are present as are dusty clay and silt intercalations.

Burial fill, directly above cranium

Large clasts of bedrock appear general absent in the deposits directly above the cranium, with most of the coarse fraction consisting of fine gravel derived from marble, sandstone/quartzite, shale/schist, speleothems and tufa. Bone fragments are more numerous than in YS and range in size from medium to coarse sand. Unlike YS, they do not exhibit induration by secondary carbonate. The micromass is generally similar to that in YS; however, unlike YS there is a high proportion of finely comminuted charcoal and organic material as well as several coarse sand-sized fragments of charcoal. Sand-sized phosphatic grains, possibly derived from hyena coprolites, are present and more numerous than in YS. Like YS, iron oxide pedofeatures are present; however, here they are expressed not as hypocoatings but as moderately impregnative matrix pedofeatures. Calcium carbonate hypocoatings are present, but appear fragmented. As in YS, coarse sand- to fine-gravel-sized fragments of laminated, dusty clay coatings and intercalations are present. Notably, this sample has a much higher proportion of void space compared to that in YS, expressed mostly and channels and vughs. The pit infilling also contains a medium gravel-sized aggregate of material resembling sediment from YS.

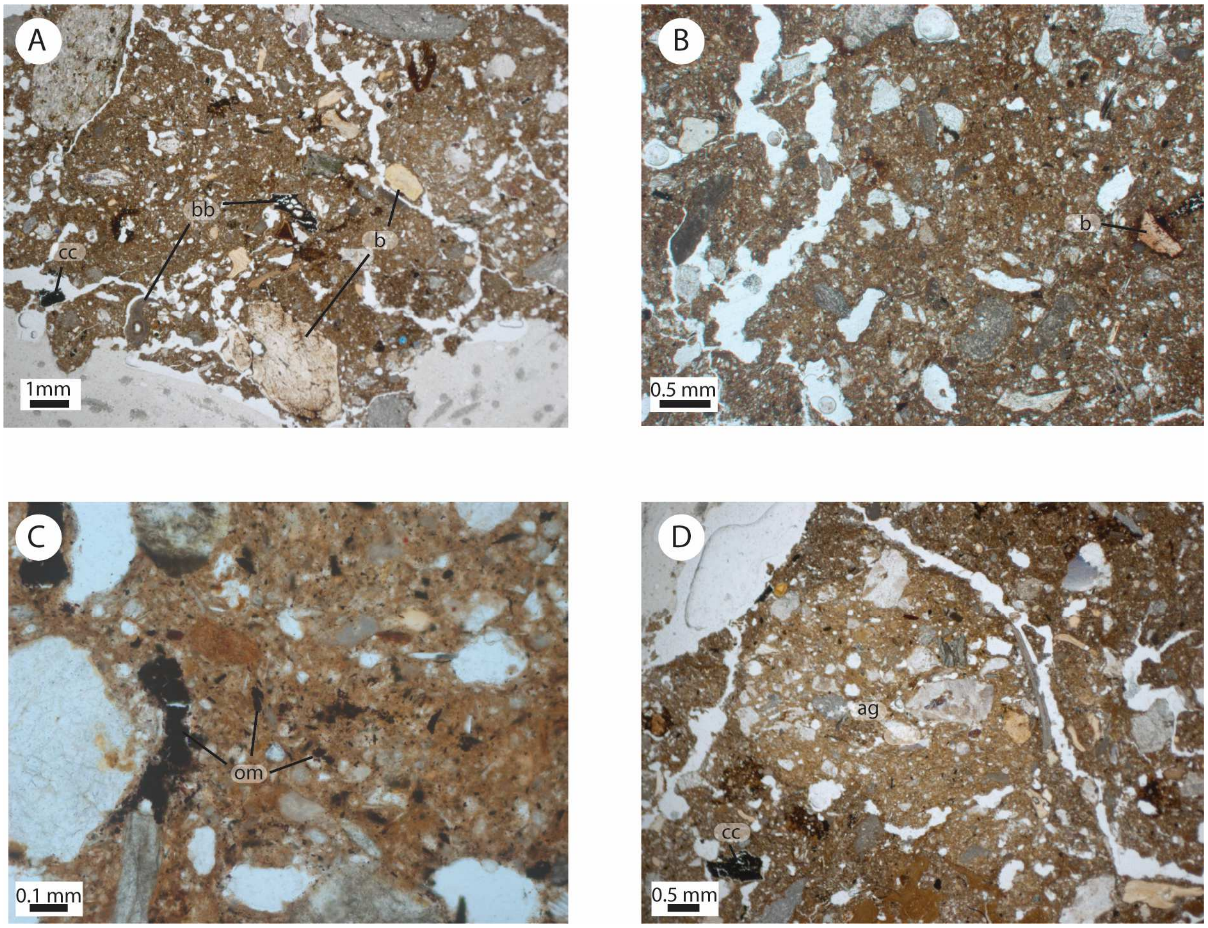

Fig. S10. Photomicrographs from thin sections of micromorphological samples collected from the burial. All are in plane poloarized light. A) From a sample of fill from the burial pit, directly above the infant cranium. The deposit here is more open, with pronounced channel microstructure. Anthropogenic components such as charcoal (cc), burnt bone (bb) and bone fragments (b) are numerous, and contrast to B) sediment from layer YS (into which the pit was dug) appears less porous and lacks significant anthropogenic material. Bone fragments are present in YS, but do not appear burnt. In the field, the pit fill appears significantly darker in color compared to the surrounding YS sediment, which is likely a result of the inclusion of anthropogenic components in the fill and also C) the inclusion of finely comminuted charcoal and organic matter (om). D) An aggregate of YS is included within the pit fill, suggesting that modification or excavation of the pit occurred shortly before infilling.

Geoarchaeological interpretation

Only a handful of micromorphological studies have been conducted on human burials (*110-114*) and even fewer have been conducted on suspected burials from Paleolithic or hunter-gatherer contexts (*115-117*). However, despite the dearth of previous work with which to compare our present data, there are several observations from the samples collected at Arma Veirana that suggest that the burial pit was intentionally excavated. In the field, the portion of the pit that was not subjected to erosion appears steep-sided, suggesting that the occupants either

modified a pre-existing depression (*118*) or intentionally excavated a pit. Additionally, the pit fill surrounding the human remains is lithologically distinct from the sediment in which the pit was dug (YS). The pit fill appears darker in the field and under the microscope this color difference is linked with a higher proportion of organic matter, charcoal and other anthropogenic components. Therefore, the pit fill itself likely derives from deposits rich in occupational debris that have not been documented through our current excavations. It is possible that these "missing" occupation horizons have been completely removed through erosion linked with the development of the rill system and the burial pit represents the preserved archive of these deposits; however, it is also possible that remnants of these occupations are still preserved further towards the back of the cave where erosive processes are less pronounced. It is also not clear if the "missing" occupation horizons were contemporaneous with the burial or if they pre-date the burial. In addition to lithological differences between the pit fill and YS, there are also marked differences in the microstructure. Compared to YS, the pit fill appears more open and has a higher proportion of void space. A more open microstructure likely suggests that the fill was dumped into the pit, which was also documented micromorphologically from an intentional burial in a pre-Islamic site of Mleiha in the United Arab Emirates (*112*). However, much of the void space in the pit fill seems to be dominated not just by vughs, but also channels, pointing to post-depositional bioturbation as a likely source for the development of at least some of the microstructure in the pit fill. Additionally, the inclusion of an aggregate of YS within the pit fill resembles the inclusion of fragmented slacking crusts also documented in burial pit fill at Mleiha (*112*). The inclusion of this aggregate at Arma Veirana suggests that modification or excavation of the pit likely happened shortly before infilling of the pit, likely implying that the pit was intentionally made for the inhumation.

Radiocarbon dating and carbon and nitrogen stable isotope analysis

All radiometric and isotopic results are shown in Tables S7 and S8. All ^{14}C dates are calibrated in OxCal v4.4 against the IntCal20 dataset (*107, 108*). A plot of the locations of the dated specimens relative to the burial contents is provided in Fig. S11. Dates obtained for the faunal bone samples by the ORAU (OxA-X-2772-12, OxA-37144, and OxA-37145) are older than the human remains. This suggests that the sediments into which the burial was dug were terminal Pleistocene in age (between 16,000 and 15,000 cal BP). These same terminal Pleistocene sediments were then used to refill the burial pit dug in the early Holocene. The dated charcoal sample (OxA-36950) was penecontemporaneous with the burial remains and likely represents surface material incorporated into the fill at the time of deposit.

Supplementary Table S7. Pretreatment and direct ^{14}C dating information for the AVH-1 specimen. Isotopic values, C:N ratios, amount of collagen extracted (%Coll, >30 kDa fraction). The results of AMS radiocarbon dating of AVH-1 specimen. $\delta^{13}C$ values are reported relative to the vPDB standard, and $\delta^{15}N$ values are reported relative to the AIR standard. The ^{14}C AMS results are calibrated using the IntCal20 (*107*) in the OxCal 4.4 (*108*) program and are rounded to the nearest 10 years

MPI code	Submitter Code*	Sample taken mg	collagen mass (mg)	%Coll	$\delta^{13}C$	$\delta^{15}N$	%C	%N	C:N	AMS Code	^{14}C Age	1σ Err	Cal BP 68.2%	Cal BP 95.4%
R-EVA 3122	human vert. 9237	133.1	5.9	4.4	-19.2	8.6	39.9	14.6	3.2	MAMS-42391	8,950	30	10,200 – 9,960	10,211 – 9,910

*Plotted find number

Table S8. Pretreatment and ^{14}C dating information for charcoal and faunal remains from the burial pit and immediate surroundings. The ^{14}C AMS results are calibrated using the IntCal20 (*107*) in the OxCal 4.4 (*108*) program and are rounded to the nearest 10 years.

OxA	Sample (plotted find #)	Material	$\delta^{13}C$	^{14}C Age ± 1σ Err	Cal BP 68.2%	Cal BP 95.4%
OxA-X-2772-12	3776	Bone (low collagen yield: 738 mg sample gave 2.6 mg collagen)	-20.15	13,010 ± 65 (accept cautiously given low collagen yield)	15,710 – 15,460	15,780 – 15,320
OxA-36950	3495	Charcoal	-27.71	9,170 ± 45	10,380 – 10,240	10,500 – 10,230
OxA-37144	3382	Bone	-19.38	13,180 ± 55	15,920 – 15,720	16,010 – 15,640
OxA-37145	3407	Bone	-19.30	12,915 ± 60	15,550 – 15,320	15,640 – 15,240

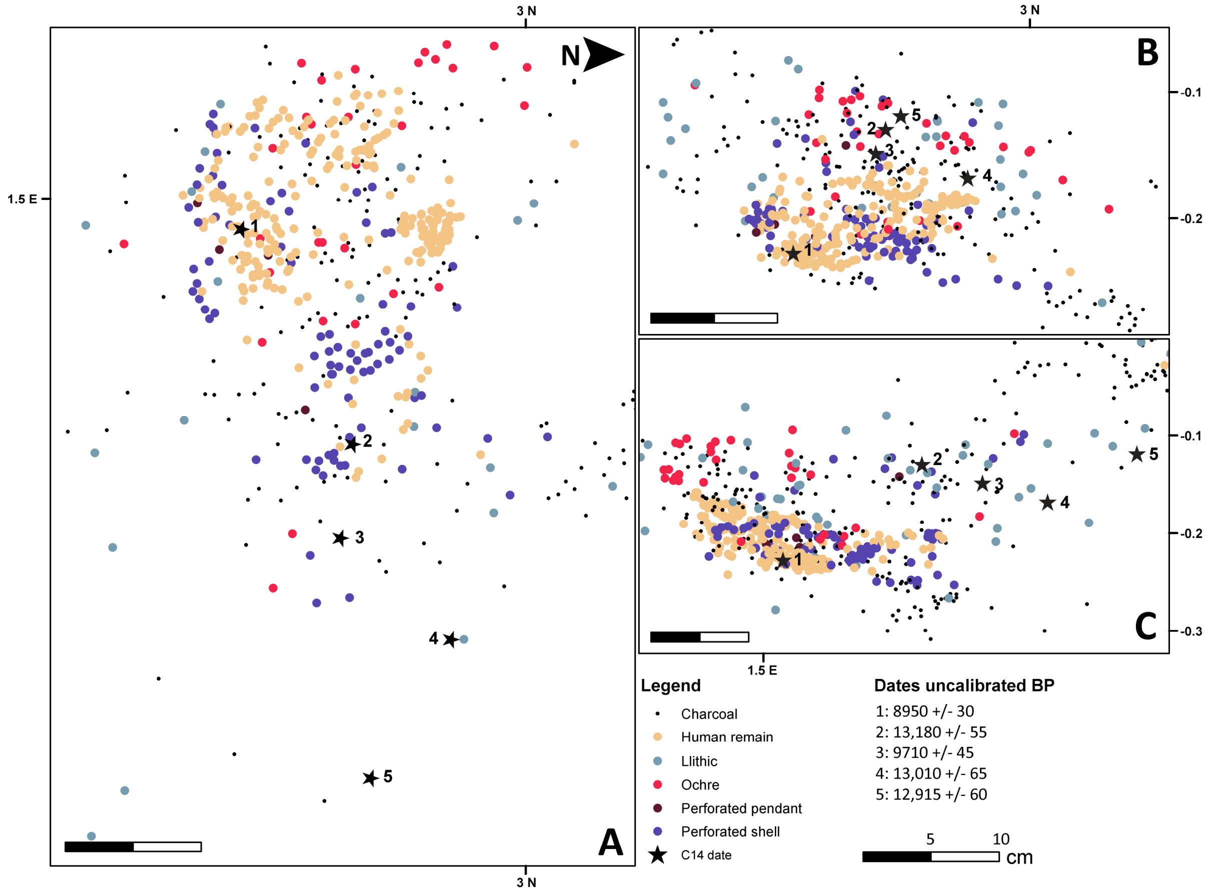

Fig. S11. Locations of radiocarbon-dated specimens relative to the burial remains. A) Plan view; B) Lateral view from the east; C) lateral view from the south. Date 1 is taken directly from the human vertebral arch. Dates 2-5 are derived from faunal material and charcoal or faunal remains composing the pit fill or deposits immediately surrounding the burial.

Skeletal remains

Most of the skeletal remains (Table S9) are readily recognizable as *Homo sapiens* based on standard osteological evaluation. However, when the burial pit was first identified in 2017, there was some question about the disposition of the bones and whether there were likely to be more human remains. An apparent cranial fragment (PF#3859) was left embedded in the western wall of the excavation quad at the end of the field season (31 July 2017). A flake of bone was sampled for ZooMS analysis ("collagen fingerprinting") to verify it as human and to help guide subsequent excavation in the 2018 season. The small fragment (~5mm) was processed for acid-soluble ZooMS analysis following the method adapted from (*119*). The resulting collagen signature contained all seven human peptid markers known in the literature (*119, 120*): 2T85 (*m/z* 1235.6), 2T43 (*m/z* 1477.7), 2T45 (*m/z* 1580.8), 2T69 (*m/z* 2115.1), 1T41 (*m/z* 2832.4), 1T55/56 (*m/z* 2885.4) and 2T67 (*m/z* 2957.4+2959.4).

SuppDataFile1-ArmaVeiranaHumanRemains.xlsx. The data file includes the following information in columns. A) Year recovered B) #pieces; C), Plotted Find #: the number assigned in the field during collection that links to the Arma Veirana database (alternatively "Sieved find" indicates small items collected during the lab screening of sediments collected from the burial excavation); D) Alt ID#: the "AVH-1" identification was assigned to indicate the infant is "Arma Veirana Hominin 1" (as of publication, all verified human remains from the burial are considered to belong to AVH-1 but only nine specimens have currently been assigned formal AltID#s with specific sub-specimen lettering such as AVH-1a, AVH-1b, etc); E) Human Status: for a few specimens, the species attribution is uncertain, but assumed to come from the burial with some possibility of being human; F) Element: skeletal part if identifiable; G -I): the Northing (y-coordinate), Easting (x-coordinate), and Elevation of the plotted find as collected by total station.

Table S9. Human and possibly human skeletal remains associated with the AVH-1 burial.

Year recovered	# pieces	Plotted Find #	Alt ID#	Human?	Element
2017	1	3055		Potential human (not probable)	Fragment
2017	1	3774		Potential human	Fragment
2017	1	3779		Potential human	Fragment
2017	4	3785		Potential human	Fragments
2017	1	3786		Potential human	Long bone shaft fragment, likely human
2017	1	3787		Potential human	Long bone shaft framgent, likely human
2017	1	3794		Potential human	Tooth
2017	1	3795	AVH-1i	Human	Right humeral shaft
2017	1	3800		Potential human	Fragment
2017	1	3831		Potential human	Fragment
2017	1	3855		Potential human	Fragment
2017	3	3859		Human (ZooMS ID)	Cranial fragment
2017	1	5391		Potential human	Rib fragment
2017	4	Sieved find		Potential human (not probable)	Fragment
2017	1	Sieved find		Potential human (not probable)	Sesamoid
2017	1	Sieved find	AVH-1a	Human	Right maxillary lateral incisor
2017	1	Sieved find	AVH-1b	Human	Left mandibular lateral incisor
2017	1	Sieved find	AVH-1c	Human	Left maxillary lateral incisor
2017	1	Sieved find	AVH-1d	Human	Left maxillary central incisor
2017	3	Sieved find		Human	Distal phalanges (n = 3)
2017	1	Sieved find		Human	Middle phalanx
2017	5	Sieved find		Human	Metacarpals or proximal phalanges (n = 5)
2017	2	Sieved find		Potential human	Long bone shaft fragment
2017	2	Sieved find		Human	Metapodial or phalangeal fragments (n = 2)
2017	5	Sieved find		Potential human	Fragment
2017	1	Sieved find	AVH-1e	Human	Left maxillary first molar (fragment)
2017	1	Sieved find	AVH-1f	Human	Right lateral mandibular incisor

Year recovered	# pieces	Plotted Find #	Alt ID#	Human?	Element
2017	1	Sieved find	AVH-1g	Human	Right maxillary first molar
2017	1	Sieved find	AVH-1h	Human	Left maxillary canine (apical fragment)
2018	1	4326		Human	Cranial fragment
2018	1	4327		Human	Fragment
2018	1	4329		Human	Cranial fragment
2018	1	4330		Human	Cranial fragment
2018	1	4612		Human	Cranial fragment
2018	1	4618		Human	Cranial fragment
2018	1	4621		Human	Cranial fragment
2018	1	4627		Human	Fragment
2018	1	4631		Human	Right frontal bone
2018	1	5033		Human	Vertebra
2018	1	5046		Human	Fragment
2018	1	5048		Human	Fragment: refits 5049
2018	1	5049		Human	Fragment: refits 5048
2018	1	5051		Human	Phalanx
2018	1	5052		Human	Fragment
2018	1	5071		Human	Fragment
2018	1	5083		Human	Fragment
2018	1	5086		Human	Fragment
2018	1	5090		Human	Long bone shaft fragment: refits 5397
2018	1	5357		Human	Vertebral fragment?
2018	1	5395		Human	Metacarpal or metatarsal
2018	1	5396		Human	Long bone shaft fragment
2018	1	5397		Human	Long bone shaft fragment: refits 5090
2018	1	5398		Human	Fragment
2018	1	5400		Human	Long bone shaft fragment
2018	1	5403		Human	Long bone shaft fragment

Year recovered	# pieces	Plotted Find #	Alt ID#	Human?	Element
2018	1	5405		Human	Fragment
2018	1	6267		Human	Cranial fragment
2018	1	6270		Human	Rib fragment
2018	1	6272		Human	Rib fragment
2018	1	6273		Human	Mandible (left corpus)
2018	1	6275		Human	Cranial fragment
2018	1	6279		Human	Rib fragment
2018	1	6280		Human	Rib fragment
2018	1	6281		Human	Rib fragment
2018	1	6284		Human	Fragment
2018	1	6285		Human	Rib fragment
2018	1	6287		Human	Fragment
2018	1	6288		Human	Left pars lateralis of occipital bone
2018	1	6289		Human	Vertebral arch fragment
2018	1	6290		Human	Rib fragment
2018	1	6291		Human	Fragment
2018	1	6292		Human	Rib fragment
2018	1	6293		Human	Rib fragment
2018	1	6294		Human	Rib fragment
2018	1	6295		Human	Rib fragment
2018	1	6296		Human	Rib fragment
2018	1	6298		Human	Fragment
2018	1	6299		Human	Fragment
2018	1	6300		Human	Rib fragment
2018	1	6302		Human	Rib fragment
2018	1	6303		Human	Fragment
2018	1	6304		Human	Rib fragment

Year recovered	# pieces	Plotted Find #	Alt ID#	Human?	Element
2018	1	6949		Human	Long bone shaft fragment (lower limb)
2018	1	6950		Human	Long bone shaft fragment (lower limb)
2018	1	6953		Human	Rib fragment
2018	1	6956		Human	Fragment
2018	1	6969		Human	Left frontal bone
2018	1	6973		Human	Rib fragment
2018	1	6978		Potential human	Fragment
2018	1	7502		Human	Cranial fragment
2018	1	7503		Human	Cranial fragment
2018	1	7504		Human	Cranial fragment
2018	1	7505		Human	Cranial fragment
2018	1	7532		Human	Fragment
2018	1	7548		Human	Cranial fragment
2018	1	7549		Human	Cranial fragment
2018	1	7551		Human	Fragment
2018	1	7552		Human	Fragment
2018	1	7553		Human	Fragment
2018	1	7554		Human	Cranial fragment
2018	1	7557		Human	Fragment
2018	1	7559		Human	Fragment
2018	1	7568		Human	Fragment
2018	1	7569		Human	Fragment
2018	1	7571		Human	Fragment
2018	1	7574		Human	Cranial fragment
2018	1	7578		Human	Cranial fragment
2018	1	9127		Human	Fragment
2018	1	9128		Human	Vertebral arch fragment
2018	1	9129		Human	Vertebra

Year recovered	# pieces	Plotted Find #	Alt ID#	Human?	Element
2018	1	9130		Human	Vertebral fragment
2018	1	9131		Human	Cranial vault and base (partial)
2018	1	9132		Human	Vertebral arch fragment
2018	1	9205		Human	Rib fragment
2018	1	9206		Human	Rib fragment
2018	1	9207		Human	Rib fragment
2018	1	9208		Human	Rib fragment
2018	1	9209		Human	Fragment
2018	1	9210		Human	Rib fragment
2018	1	9211		Human	Rib fragment
2018	1	9212		Human	Rib fragment
2018	1	9213		Human	Rib fragment
2018	1	9214		Human	Fragment
2018	1	9215		Human	Vertebral arch fragment
2018	1	9216		Human	Vertebral arch fragment
2018	1	9217		Human	Vertebral arch fragment
2018	1	9218		Human	Vertebral arch fragment
2018	1	9219		Human	Vertebral arch fragment
2018	1	9220		Human	Vertebral centrum
2018	1	9221		Human	Vertebral arch fragment
2018	1	9222		Human	Vertebral fragment
2018	1	9223		Human	Vertebral arch fragment
2018	1	9224		Human	Vertebral arch fragment
2018	1	9225		Human	Cranial fragment
2018	1	9226		Human	Vertebral arch fragment
2018	1	9229		Human	Cranial fragment
2018	1	9236		Human	Vertebral fragment
2018	1	9237		Human	Vertebral fragment

Year recovered	# pieces	Plotted Find #	Alt ID#	Human?	Element
2018	1	9238		Human	Vertebral arch fragment
2018	1	9239		Human	Vertebral arch fragment
2018	1	9240		Human	Vertebral arch fragment
2018	1	9241		Human	Right pars lateralis of occipital bone
2018	1	9242		Human	Vertebral arch fragment
2018	1	9243		Human	Left mandibular first molar
2018	1	9244		Human	Cranial fragment
2018	1	9245		Human	Right scapula and nonidentifiable fragments
2018	1	9247		Human	Vertebral arch fragment
2018	1	9248		Human	Fragment
2018	1	9249		Human	Fragment

Dental analysis results

Virtual histology

The neonatal line (NL), marking the birth event, is well visible in all the virtual histological sections at medium spatial resolution on both the buccal and lingual sides. DSRs were measured in the high spatial resolution virtual slices of the AVH-1d and AVH-1g in the third cervical of the crown, both in prenatal and postnatal enamel, by counting daily increments along prisms segments of known length. The DSR values are reported in Table S10. The amount of enamel secreted and mineralized after birth, and consequently the age-at-death of AVH-1, was calculated in the best preserved enamel portions dividing the prisms' lengths, from the NL to the outer enamel, by the postnatal DSR (for the upper central incisor AVH-1c the DSRs of the lateral upper incisor AVH-1c was used). The results are highly consistent in the three crowns, giving an age-at-death for the child between 40-50 days (Table S11). A correction of 7 minimum and 15 maximum days for the immature enamel (*17*), lost post-depositionally, was applied.

Moreover, two accentuated lines (ALs) are present in the prenatal enamel of all the examined teeth, well discernible in the medium spatial resolution virtual sections on both the buccal and lingual side. The amount of enamel formed between the ALs and birth and the corresponding chronologies has been calculated dividing the prism lengths, measured from the AL to the NL, by the prenatal DSR. Results (Table S12) are highly consistent for the three crowns, suggesting that during pregnancy two severe physiological stress episodes affected the fetus and/or the mother at ca 1 month (27 days) and ca 1.5 months (46 days) before birth.

Table S10. Enamel daily secretion rates in the cervical portion of two dental deciduous crowns of AVH-1, estimated by mean of virtual histology on high spatial resolution reformatted images.

Sample ID	Tooth type	Enamel portion	DSR
AVH-1d	Upper Central Incisor	prenatal	4.9
AVH-1d	Upper Central Incisor	prenatal	5.1
AVH-1d	Upper Central Incisor	prenatal	5.1
AVH-1d	Upper Central Incisor	postnatal	4.7
AVH-1g	Upper First Molar	prenatal	5.1
AVH-1g	Upper First Molar	prenatal	4.9
AVH-1g	Upper First Molar	prenatal	4.9
AVH-1g	Upper First Molar	prenatal	4.8
AVH-1g	Upper First Molar	prenatal	4.8
AVH-1g	Upper First Molar	postnatal	5.4
AVH-1g	Upper First Molar	postnatal	4.9
AVH-1g	Upper First Molar	postnatal	5.2
AVH-1g	Upper First Molar	postnatal	4.5
AVH-1g	Upper First Molar	postnatal	4.8

Table S11. AVH-1's age-at-death estimate by mean of virtual histology of three dental deciduous crowns. Results are based on medium spatial resolution reformatted images.

Sample ID	Tooth type	Age-at-death estimate (days)	Age-at-death with 7 days correction factor (days)	Age-at-death with 15 days correction factor (days)
AVH-1d	Upper Central Incisor	36	43	51
AVH-1c	Uppel Lateral Incisor	31	38	46
AVH-1g	Upper First Molar	33	40	48
Mean of three teeth		**33**	**40**	**48**

Table S12 Chronology of two physiological stress episodes during AVH-1's mother pregnancy estimated by mean of virtual histology of three dental deciduous crowns. Results based on medium spatial resolution reformatted images. AL1 = most apical accentuated Line; AL2 = most cervical accentuated line.

Sample ID	Tooth type	AL1	AL2
AVH_1d	Upper Central Incisor	-47	-29
AVH_1c	Uppel Lateral Incisor	-44	-24
AVH_1g	Upper First Molar	-46	-27
Mean of the three teeth		**-46**	**-27**

Amelogenin-based sex estimation

Both the ion chromatograms and the Mascot searches identified the presence of AMELX within AVH-1's tooth enamel (Fig. 4A and Fig. S12). Contrariwise, no AMELY unique peptide was found. This suggests that AV1 was possibly a female individual. False positive female identification is rare but possible; e.g. in the case of partial lacks of the amino acid sequence (*56*). Other tooth-related proteins were also confidently identified as ameloblastin and collagen type I. Mascot search indicated the presence of fetuin_A, a glycoprotein abundant in the fetal plasma. No modern contaminant was identified through the cRAP database.

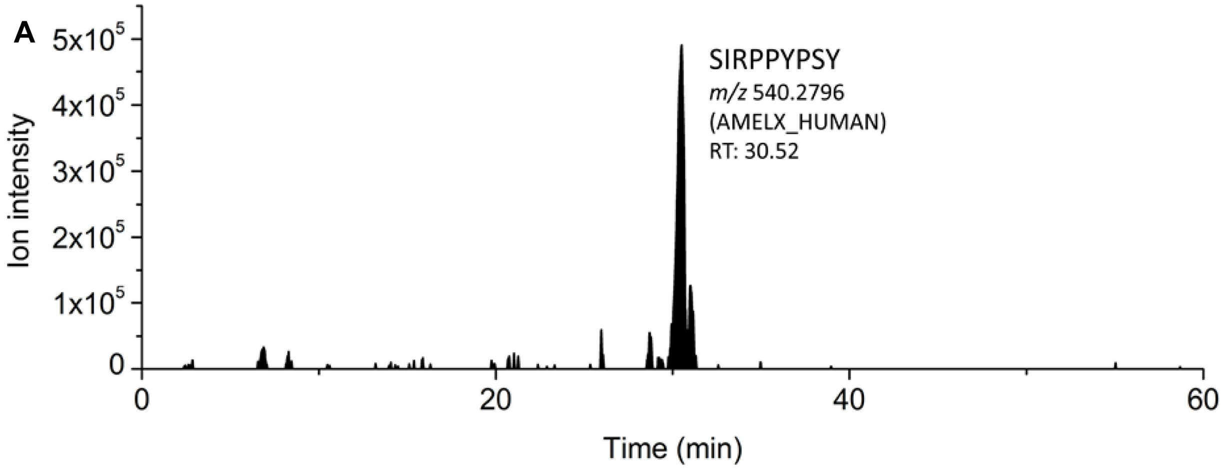

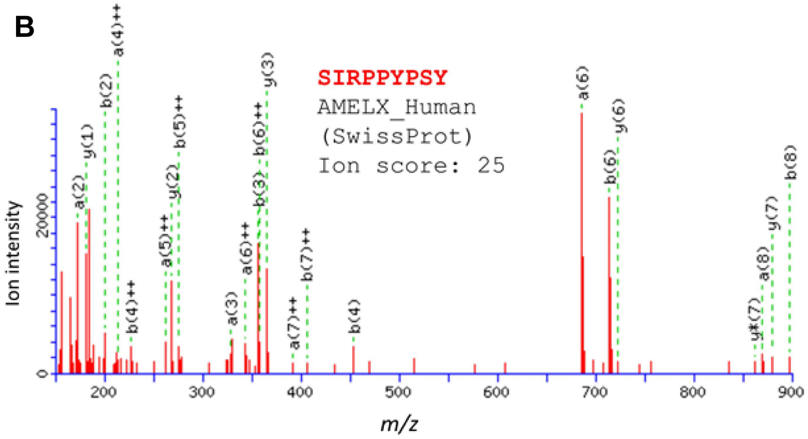

C

Protein (ID)	Protein score	Pep (sig)
AMELX (Q99217)	187	30
CO1A2 (P08123)	112	7
CO1A1 (P02452)	101	10
AMBN (Q9NP70)	87	8
FETUA (P02765)	91	8

Fig. S12. Summary of amelogenin-based sex estimation and proteomic analyses. (A) Ion chromatograms of peptide SIRPPYPSY (AMELX, 540.2796 *m/z*) as in (*22*); peptide SM(ox)IRPPY (AMELY) has not been detected, as well as any other AMELY peptide. (B) Fragmentation spectrum of peptide SIRPPYPSY from the Mascot search. (C) Summary table of identified proteins, with the number of significant peptides observed and the protein score.

Ancient DNA Results

DNA was extracted (*57*) from 9.5 mg and 8.1 mg of bone powder removed from the vertebral fragment of AVH-1 and converted into single-stranded DNA libraries (*58*). Following the enrichment for human mitochondrial DNA (mtDNA) (*60*), we recovered a total of 212,145 unique mitochondrial DNA fragments from both libraries (Table S4). The frequency of cytosine (C) to thymine (T) substitutions at the ends of these fragments were 45.2% and 34.6%, respectively (Table S3), indicating the preservation of deaminated ancient DNA molecules in the specimen (*67, 75*). Using 59,824 deaminated fragments, we reconstructed a full mitochondrial genome (*68*) of the AVH-1 individual, at an average 199-fold coverage. Based on a phylogenetic tree built within a Bayesian framework using the AVH-1 mtDNA together with mtDNAs from 54 present-day and 52 radiocarbon dated ancient human individuals (Table S6, Fig. S13), we estimated a molecular age of 9,774years BP (95% highest posterior density (HPD): 1,853-16,662). A comparison of sequence coverage of the X chromosome and the autosomes (*85*)(*6*) (Fig. 4B) obtained through shallow shotgun sequencing shows that the AVH-1 individual was female, congruent with results from the proteomics analysis.

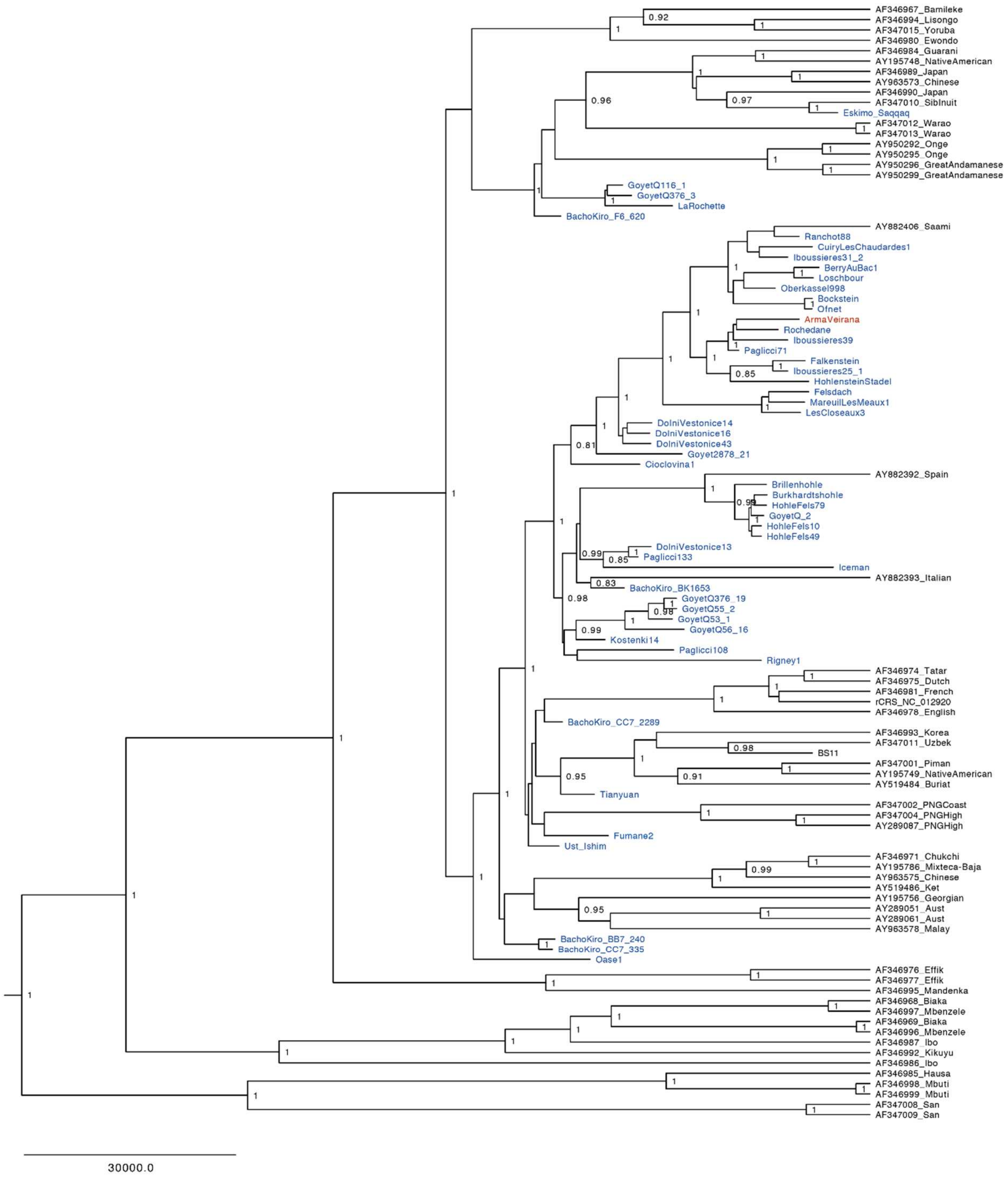

Figure S13 Bayesian phylogenetic tree relating AVH-1 mtDNA to 54 present-day and 52 radiocarbon dated ancient humans. The AVH-1 specimen is highlighted in red. Other ancient human mtDNA genomes are highlighted in blue. Present-day human mtDNAs are in black. The posterior probabilities are denoted above the branches. The mtDNA of Vindija 33.16 was used to root the tree (not shown).

<u>Burial artifacts: shell beads and pendants</u>

Goods in direct association with the human remains (i.e., at the same elevation and < 5cm from the nearest bone) comprise 66 perforated *Columbella rustica* and 3 *Glycymeris* sp. pendants. An additional 27 *C. rustica*, one *Turritella* sp., and one *Glycymeris* sp. pendant were found in the burial fill, in the burrow, or washed away in adjacent squares. Out of this total of 98 perforated shells, 89 were piece-plotted with the total station, six were found in sieved material, and three remain underneath the cranium. Four perforated *C. rustica* were put aside for dating and therefore remain unstudied. The average length and width of the 86 *C. rustica* for which we have metrics are 14.03mm and 8.42mm, respectively, with an average perforation radius of 2.56mm. These measurements and the 3D coordinates for all piece-plotted shells are provided in Supplementary data file 2. Most specimen have only one perforation, located across from their natural aperture. In two cases (specimens #3856 and 6966), a second perforation is found further in the body whorl. Ochre residues are found on most perforated shells, but in small quantities. Most perforated *C. rustica* have mild to intense use-wear on the left and top left portion of the perforation as well as the bottom of their siphon canal. A few shells are extremely worn and missing most of their spires, suggesting that they had been worn for an extended period before their discard.

The one *Turritella* sp. shell was found in the sediment above the human remains, at the same elevation as one of the older dates obtained on the burial, which suggests that it may have been part of the burial fill rather than the burial itself. The shell's spire is broken, and the breakage is rounded, suggesting that the shell was worn for a certain amount of time after it broke. The shell is 17.65mm long and 9.53mm wide and it has two elongated perforations in its body whorl.

The preliminary reconstruction experiment determined that the pendants were made on beach-worn pieces of *Glycymeris* sp. The pendants have a roughly similar shape and size. Their mean average length, width, and thickness are 33.33mm, 23.19mm, and 4.10mm respectively. All pendants are perforated only once, the perforations have an average radius of 1.75mm and are located on one side of the elongated axis. Three out of four perforations have a biconical profile, suggesting that they were perforated using bilateral drilling. The pendants have heavy use-wear on their ventral surface, suggesting that they were worn with their dorsal side up for a considerable amount of time before being discarded.

SuppDataFile2-ShellOrnamentData.xlsx. The data file includes the following information in columns. A) PF#: the number assigned in the field during collection that links to the Arma Veirana database (alternatively "Sieved find" indicates small items collected during the lab screening of sediments collected from the burial excavation; as of publication, three specimens are still attached to the underside of the cranium, which was removed as a block with some sediment still adhering); B) Lot#: a number assigned in the field to designate a subarea excavated within a quadrant of a specific square (all lots here considered to be part of the burial); C-E): Northing (y-coordinate), Easting (x-coordinate), and Elevation (z-coordinate) of the plotted find as collected by total station); F) Location: the location on the body or the *ex situ* context in which the specimen was recovered; G) Taxon: the species from which the shells are derived; H) Length: maximum length of the specimen (mm); I) Width (mm): maximum width of the specimen; J) Perf length (mm): the maximum width of the perforation (the hole drilled into the specimen, presumably used for stringing).

Burial artifacts: bird of prey claw (PF# 6877)

The pedal avian phalanx (Fig. S14) was determined to be that of an eagle owl (*Bubo bubo*). It is complete and shows infiltration of manganese oxides and punctiform corroded groove in dorsal bone surface at the insertion of the terminal keratinous claw (keratin case). The anthropic traces are localized laterally the perimeter of the proximal articular facet (Fig. S14, subpanels 1-2). The localized semicircular cutmark suggests the cutting of the medial or lateral extensor tendons and ligaments. On the articular margin, partial tearing and/or peeling fresh bone fracture to the cortical bone tissue is clearly visible, obliquely crossing the surface of the articular facet, near the cutmark (Fig. S14).

The claw also preserves modifications related to use: they are polishes and narrow striations. Polished zones are mostly extended on a side of the phalanx and involve all surfaces except for the articular one (Fig. S15). In particular, the distal extremity of the claw, which is broken, appears slightly rounded and polished. This wear could be due to punctual crushing of the keratin case with the bone claw, in association with a forced movement, but we cannot rule out taphonomic causes. These traces cannot have occurred during the lifetime of the raptor (*92*).

The macro-traces on the claw are certainly associated with the same purpose: disarticulation. The claw also shows dully developed surface modifications related to ornamental use. The minimal development of wear could suggest the Arma Veirana pendant was used only for a brief time (*121, 122*). The ornamental use of claw elements due to their length and curvature, and possibly their suspension in different ways, is a common feature in the ethnographic records. This interpretation of pedal phalanges' use as pendants, suspended in isolation or still connected to the entire foot has also been suggested at Middle Palaeolithic (*92, 123*), Châtelperronian (*124*), and Aurignacian (*125*) sites, and an over-representation of raptor feet bones is recorded for recent periods of the Upper Palaeolithic (*126, 127*). The symbolic use of bird prey remains is also clearly attested in more recent periods from voluntary deposition associated with human burials (*128*).

Whether or not this specimen is directly associated with the infant burial is uncertain given it was located in a small secondary pit directly adjacent to the border of the AVH-1 pit. If not, it may indicate the presence (or former presence) or other burials in the cave.

Included as separate supplementary files are the following 3D polygon models of select artifacts.

- **ArmaVeirana-PF3484-Glycymeris-pendant.ply.** Plotted find #3484 perforated shell pendant made from *Glycymeris* sp.
- **ArmaVeirana-PF3854-Glycymeris-pendant.ply.** Plotted find #3854 perforated shell pendant made from *Glycymeris* sp.
- **ArmaVeirana-PF3858-Glycymeris-pendant.ply,** Plotted find #3858 perforated shell pendant made from *Glycymeris* sp.
- **ArmaVeirana-PF6877-Bubo-talon.ply,** Plotted find #6878 pendant made from an eagle owl (*Bubo bubo*) talon (pedal distal phalanx).
- **ArmaVeirana-PF9228-Glycymeris-pendant.ply.** Plotted find #9228 perforated shell pendant made from *Glycymeris* sp.

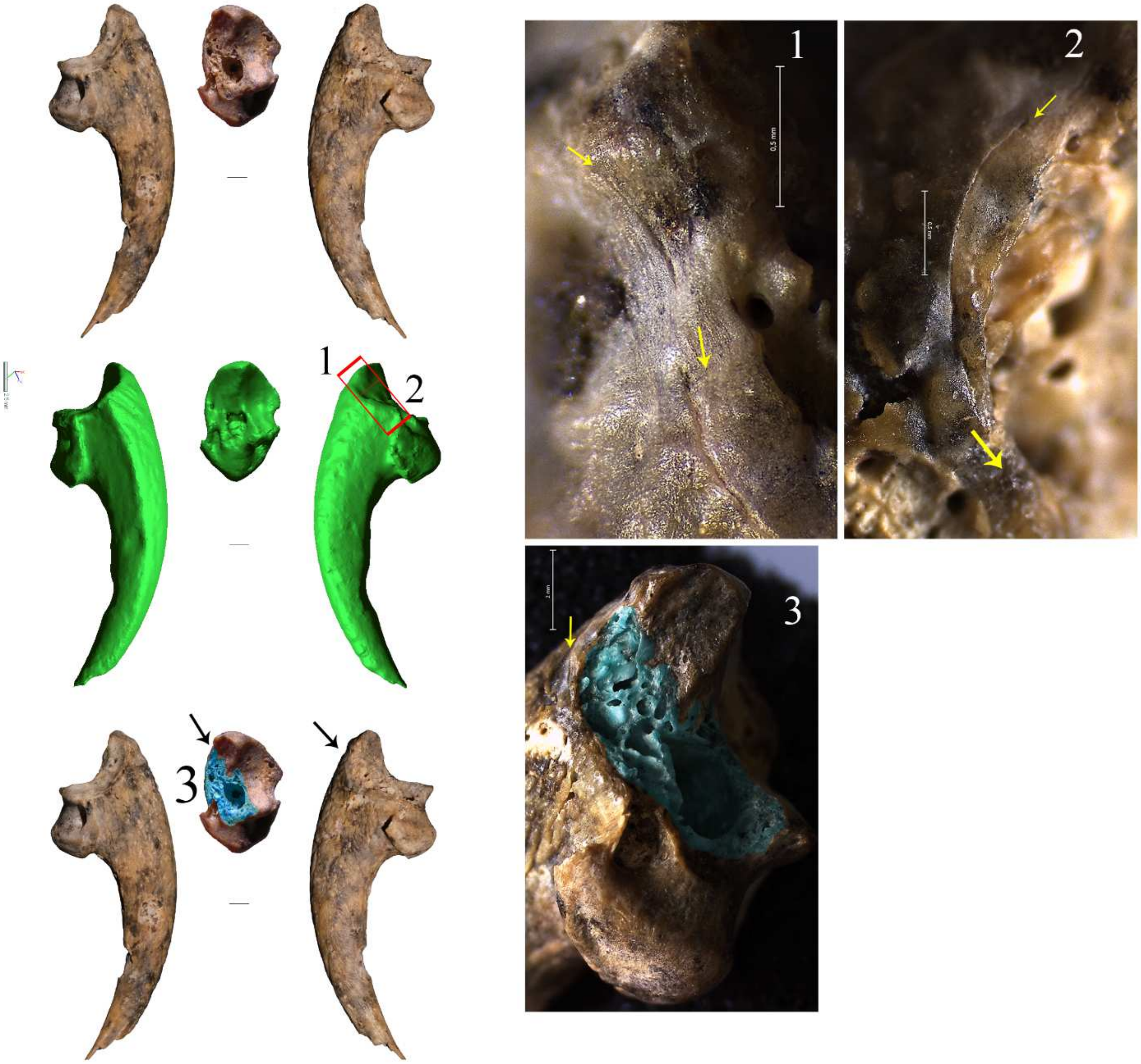

Fig S14. Complete terminal pedal phalanx of an eagle owl (*Bubo bubo*) (PF#6877). The numbers and the black arrows 1 – 3, indicate the localization, orientation of the recognized anthropic traces. The yellow arrows in the stereomicroscope close-up photos, indicate the localization of traces and their orientation. 1 – 2: semicircular cutmark; 3 and transparent blue area: wrenching and peeling fresh bone fracture.

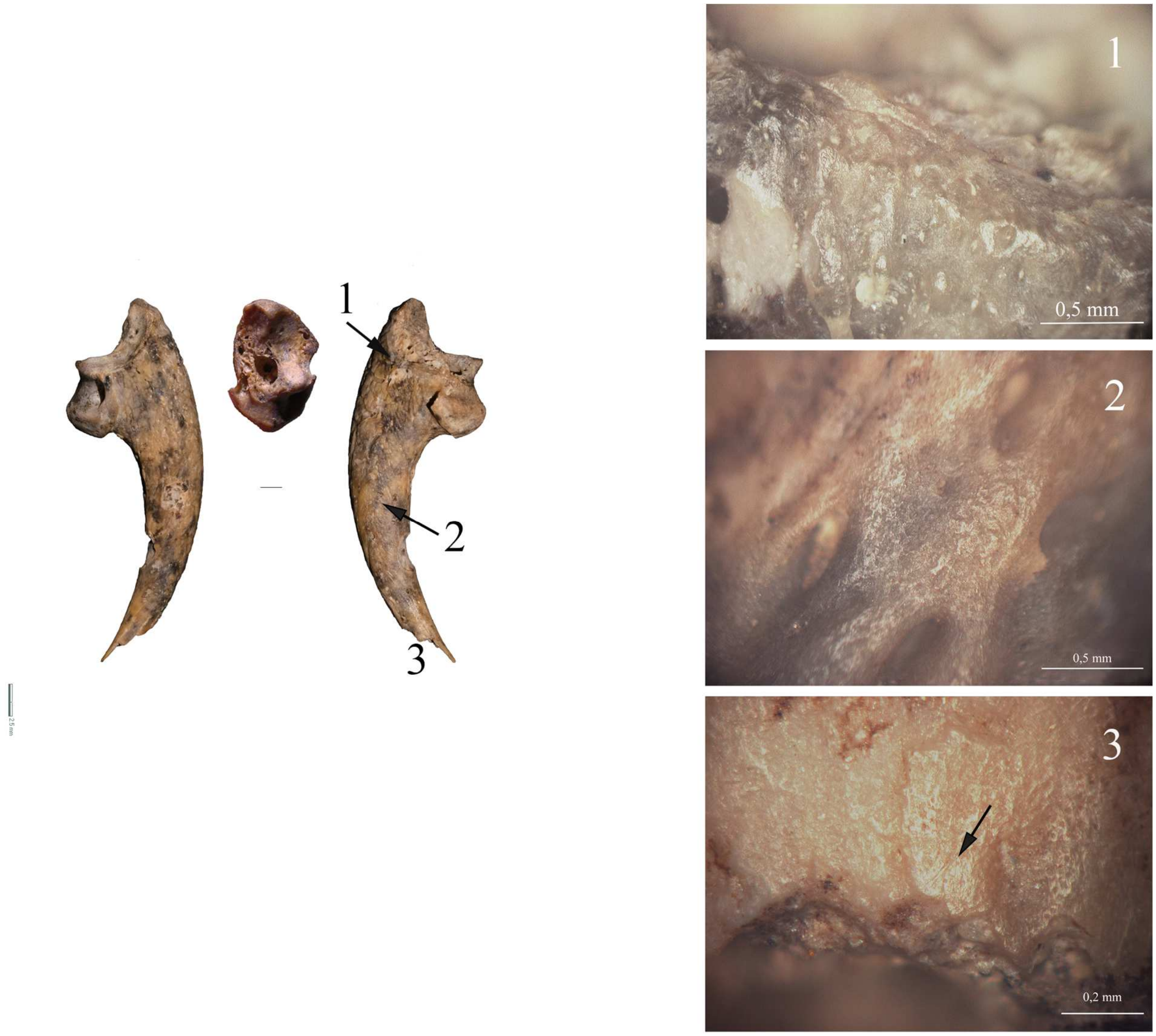

Fig. S15. Use wear detected on the eagle owl phalanx. The numbers (1 – 3) and the black arrows indicate the localization of the recognized traces. 1 – 2 Dully polished areas; 3 Rounding and polishing on the distal extremity of the claw.

Lithics

The burial pit yielded 19 chipped stone tools made on radiolarite and on different type of flint (Table S13). Sixteen of these artifacts consist of tiny fragments deriving from knapping or retouching activities. Retouched tools are very rare: two armatures and a retouched laminar flake (Figs. S16 and S17). The two armatures consist of a fragmentary backed and obliquely-truncated piece and of the mesial portion of a double-backed blade (or point) (Fig. S17/2-3). The laminar flake (PF#4587) presents a marginal, direct retouch on the two edges and has been manufactured on a gray Jurassic-Cretaceous flint, known from outcrops in Eastern Liguria ("Calcari a Calpionelle") and in Emilia ("Maiolica" Formation), both of which are over 100 kilometers distant as the crow flies (*129, 130*). PF#4587 was collected very close to the human cranium and it is thus likely, for its specificity, the only lithic piece that can likely be interpreted as a grave good.

None of these artifacts are strongly diagnostic of Mesolithic affinities. The two armatures are also found within the regional Late Epigravettian hunter-gatherer tool-kit (*25, 131*), while the only possible Sauveterrian-like piece, from a typological point of view, is a very small fragmentary double-backed point, on imported grey flint, coming from fill deposit from the top of the burial pit (Fig. S17/1). All the artifacts (with the likely exception of the laminar flake) were displaced by the excavation of the burial pit into sediments dated to approximately 16-15 ka cal BP (Table S8), indicating a Late Epigravettian age for the deposits into which the burial pit of AVH-1 was dug. Furthermore, microburins are absent, despite being the most common lithic technological byproduct during the Late Epigravettian after 16-15 ka and of the Mesolithic in Italy and Provence-Liguria (*25*). This argues against a direct association between the majority of these lithics and the burial.

The laminar flake presents very marginal retouch, possibly resulting from its use as a scraper or knife (Fig. S16). If, as we propose, it can be really associated to the grave, its presence is intriguing and it may be symbolically linked to the act of the burial or have been even used in some birth rite. Ethnographic data from Australia, for example, document that "the knives", also made from stone, were "used for various tasks such as cutting meat and severing a newborn infant's umbilical cord" (*132*) (p. 70). Although we cannot have absolute certainty about its association with the burial, the typological peculiarity of this artifact, the raw material from which it was made and the fact that it was found near the skull, makes this hypothesis plausible.

Table S13. Lithics associated with the AVH-1 burial fill.

Plotted Find #	Description	Dimensions (mm)	Raw material
4587	Blade-like flake with marginal, direct, bilateral retouch	31-11-3	Gray flint (Calcari a Calpionelle or Maiolica formation
3792	Fragment of backed and obliquely-truncated piece	13-9-5	Pinkish-brown flint, burnt
7540	Fragment of double-backed piece	11-6-2.5	Pinkish-brown flint, matt
From sieving	Debris	9-7-1	Redish-brown radiolarite
From sieving	Debris	8-4-1	Redish-brown radiolarite
From sieving	Debris	6-5-1	Redish-brown radiolarite
From sieving	Debris	5-4-1	Redish-brown radiolarite
From sieving	Debris	6-3-1	Redish-brown radiolarite
From sieving	Debris	3-3-1	Redish-brown radiolarite
From sieving	Debris	13-3-2	Gray flint
From sieving	Debris	2.5-2-1	Light brown flint
From sieving	Debris	3-1.5-1	Light brown flint with whitish speckles (possibly radiolarite)
From sieving	Debris	3-2.5-1	Light brown flint with whitish speckles (possibly radiolarite)
From sieving	Debris	5-4-1	Light brown flint with whitish speckles (possibly radiolarite)
3790	Debris	10-7-1	Light grayish-brown flint, burnt
From sieving	Debris	2-2-1	Brown flint with whitish speckles (possibly radiolarite)
From sieving	Debris	3-2-1	Brown flint with whitish speckles (possibly radiolarite)
From sieving	Debris	21-15-5	Yellowish-brown flint
From sieving	Debris	4-2-1	Pinkish flint

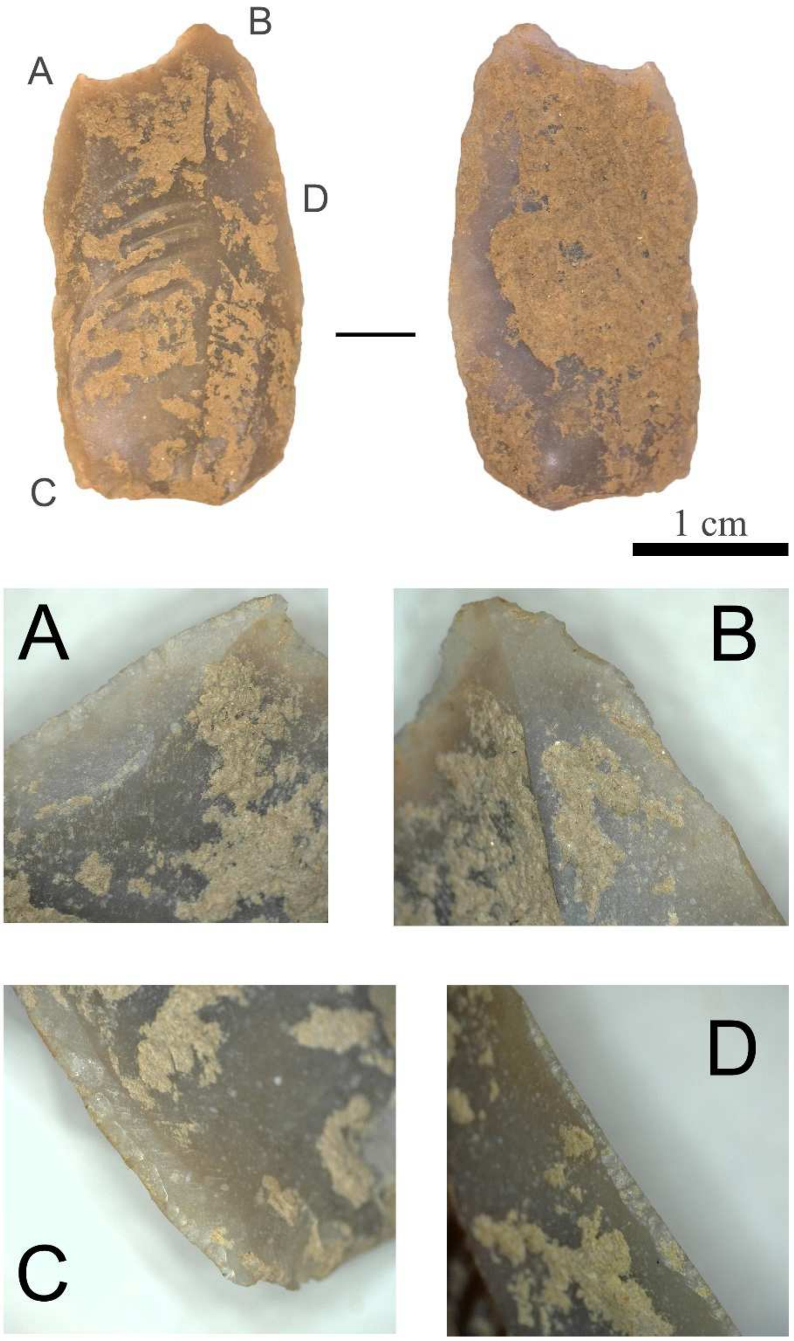

Fig. S16. Retouched laminar flake (PF#4587) from the AVH-1 burial pit. A-D) Magnifications of the marginally retouched edges. This piece is the only lithic that can be confidently interpreted as a grave good given its close spatial association to the skeleton.

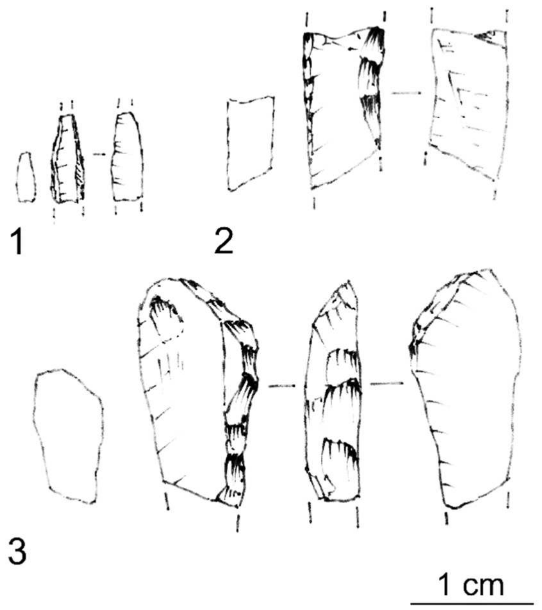

Fig. S17. Retouched lithic pieces from the AVH-1 burial pit. 1) Fragmentary double-backed point (Sauveterrian point) (from the removed deposit); 2) fragmentary backed and obliquely-truncated piece (PF#3792); 3) Mesial portion of a double-backed blade (or point).

Faunal Analysis

Fauna recovered from the burial pit represent backfill. Most of the faunal remains can only be identified as mammalian (Table S14) as they are highly fragmented. Of fragments identifiable to a mammalian group, most come from taxa likely to have been prey and were almost certainly not ceremonial in nature (Table S14). In fact, a small percentage of the faunal remains in the burial preserve butchering marks indicating they were consumed as food (Table S15). Carnivore tooth marks were found on a small percentage of remains (Table S15) indicating that carnivores played some role in modifying and/or accumulating the faunal remains that ended up in the burial fill. The remains of young juvenile *S. scrofa* including a mandible and three teeth were recovered in sediments close to the burial but not directly related to it. One fragment likely belonging to a bird was found with other faunal bones in the burial, but it is poorly preserved and not identifiable to taxon.

The faunal remains from the burial fill suggest a wooded environment based on the presence of wild boar (*133*). However, these fauna date to the terminal Pleistocene (Table S8) and are therefore 5000-6000 years older than the AVH-1 human remains dating to the early Holocene (Table S7). Therefore, the fauna are unlikely to accurately reflect the local environment at the time of inhumation. Future efforts at Arma Veirana and sites in the vicinity should focus on identifying cultural deposits that are truly contemporaneous with the burial itself to further understand the environments of the Ligurian pre-Alps during the earliest Holocene when the population that buried AVH-1 was occupying the area.

Table S14. Fauna identified in the burial fill.

Identification	Number of Identifiable Specimens (NISP)	Frequency (%)
Artiodactyla	2	1.942
Avian	5	4.854
Bos/Bison	1	0.971
Bovid	1	0.971
Capra ibex	1	0.971
Non-ID mammal	79	76.699
Non-ID	10	9.709
Sus	4	3.883
**Total**	**103**	

Table S15. Surface modifications on faunal bones.

Modification type	# of bones	Frequency*
Percussion marks	2	2.0
Percussion notches	6	5.9
Cut marks	3	3.0
Carnivore tooth marks	4	4.0
No modification	86	85.1
**Total # of bones**	101	

***As a percentage of the total number of bones.**

SUPPLEMENTARY REFERENCES

arcodes in excavation projects: examples from Mossel Bay (South Africa) and Roc de Marsal (France). *The SAA Archaeological Record* **7**, 33-38 (2007).

Kuester, Visualizing high resolution three-dimensional and two-dimensional data of cultural heritage sites. . *Mediterr Archaeol Archaeom* **14**, 93-100 (2014).

A new method of analyzing and documenting micromorphological thin sections using flatbed scanners: Applications in geoarchaeological studies9+14. *Geoarchaeology* **17**, 305-313 (2002).

. Vandermeulen, Documenting archaeological thin sections in high-resolution: A comparison of methods and discussion of applications. *Geoarchaeology* **34**, 100-114 (2019).

Soils and micromorphology in archaeology. (Cambridge University Press, Cambridge, 1989).

Guidelines for analysis and description of soil and regolith thin sections. (Soil Science Society of America, Madison, 2003).

et al., Enhanced and flexible software for x-ray computed tomography at the Italian Synchrotron Radiation Facility Elettra. *Fundam Inform* **141**, 233-243 (2015).

. Wilkins, Simultaneous phase and amplitude extraction from a single defocused image of a homogeneous object. *J Microsc* **206**, 33-40 (2002).

et al., Fiji: an open-source platform for biological-image analysis. *Nat Methods* **9**, 676-682 (2012).

et al., The IntCal20 Northern Hemisphere radiocarbon age calibration curve (0–55 cal kBP). *Radiocarbon* **62**, 725-757 (2020).

Radiocarbon **51**, 337-360 (2009).

ies identification by analysis of bone collagen using matrix-assisted laser desorption/ionisation time-of-flight mass spectrometry. *Rapid Commun Mass Spectrom* **23**, 3843-3854 (2009).

, The geoarchaeology of Mycenaean chamber tombs. *J Archaeol Sci* **39**, 2722-2732 (2012).

ødsrud, Integrated microstratigraphic investigations of coastal archaeological soils and sediments in Norway: The Gokstad ship burial mound and its environs including the Viking harbour settlement of Heimdaljordet, Vestfold. *Quat Int* **315**, 131-146 (2013).

et al., Late pre-Islamic burials at Mleiha, Emirate of Sharjah (UAE). *Arabian Archaeology and Epigraphy* **25**, 175-185 (2014).

pened early Bronze Age inhumation grave in Austria: The soil thin section analyses. *J Archaeol Sci Rep* **10**, 791-809 (2016).

et al., Micromorphological and chemical investigation of late-Viking age grave fills at Hofstaðir, Iceland. *Geoderma* **306**, 183-194 (2017).

et al., Testing the roc de Marsal Neandertal “burial” with geoarchaeology. *Archaeol Anthropol Sci* **9**, 1005-1015 (2017).

116. X. S. Villagran, A. Strauss, C. Miller, B. Ligouis, R. Oliveira, Buried in ashes: Site formation processes at Lapa do Santo rockshelter, east-central Brazil. *J Archaeol Sci* **77**, 10-34 (2017).
117. E. Pomeroy *et al.*, Issues of theory and method in the analysis of Paleolithic mortuary behavior: A view from Shanidar Cave. *Evol Anthropol* **29**, 263-279 (2020).
118. W. Rendu *et al.*, Evidence supporting an intentional Neandertal burial at La Chapelle-aux-Saints. *Proc Natl Acad Sci U S A* **111**, 81-86 (2014).
119. M. Buckley, S. W. Kansa, Collagen fingerprinting of archaeological bone and teeth remains from Domuztepe, South Eastern Turkey. *Archaeol Anthropol Sci* **3**, 271-280 (2011).
120. S. Brown *et al.*, Identification of a new hominin bone from Denisova Cave, Siberia using collagen fingerprinting and mitochondrial DNA analysis. *Sci Rep* **6**, 253559 (2016).
121. Y. Taborin, in *Paris, Ed. CNRS. Suppément à Gallia Préhistoire* (1993), vol. 29.
122. F. D'Errico, in *Traces et function:les gestes retrouvées. ERAUl 50,* P. C. Anderson, S. Beyries, M. Otte, Eds. (1993), pp. 177-188.
123. M. Romandini *et al.*, Convergent evidence of eagle talons used by late Neanderthals in Europe: a further assessment on symbolism. *PLoS One* **9**, e101278 (2014).
124. A. Rodriguez-Hidalgo *et al.*, The Chatelperronian Neanderthals of Cova Foradada (Calafell, Spain) used imperial eagle phalanges for symbolic purposes. *Sci Adv* **5**, eaax1984 (2019).
125. V. Laroulandie, E. Morin, M.-C. Soulier, J. C. Castel, Bird procurement by humans during the Middle and early Upper Paleolithic of Europe: New data for the Aurignacian of southwestern France. *Quat Int* **543**, 16-24 (2020).
126. T. Simmons, D. Nadel, The avifauna of the Early Epipalaeolithic site of Ohalo II (19,400 B.P.), Israel: species diversity, habitat and seasonality. *Int J Osteoarchaeol* **8**, 79-96 (1998).
127. S. L. Kuhn *et al.*, The last glacial maximum at Meged Rockshelter, Upper Galilee. *Israeli J Prehist Soc* **34**, 5-47 (2004).
128. L. Grosman, N. D. Munro, A. Belfer-Cohen, A 12,000-year-old Shaman burial from the southern Levant (Israel). *Proc Natl Acad Sci U S A* **105**, 17665-17669 (2008).
129. F. Negrino, E. Starnini, Patterns of lithic raw material exploitation in Liguria from the Palaeolithic to the Copper Age. *Préhistoire du Sud-Ouest* **Supplément n.5**, 235-243 (2003).
130. G. Porraz, P. Simon, A. Pasquini, Identité technique et comportements économiques des groupes proto-aurignaciens à la grotte de l'Observatoire (Principauté de Monaco). *Gallia Préhistoire* **52**, 33-59 (2010).
131. A. Palma di Cesnola, in *Actes du colloque international «La position taxonomique et chronologique des industries à pointes à dos autour de la méditerranée européenne», Siena 3-6 novembre 1983, Rivista di scienze preistoriche,* A. Palma di Cesnola, Ed. (1983), vol. 38, pp. 301-318.
132. S. M. Nelson, *Gender in archaeology: analyzing power and prestige*. (AltaMira Press, Lanham, 1997).
133. T. Abajgar, G. del Barrio, J. R. Vericad, Habitat preference of wild boar (*Sus scrofa* L., 1758) in a mediterranean environment. Indirect evaluation by signs. *Mammalia* **58**, 201-210 (1994).
